# Effect of valve opening on starting performance of pump as turbine

**DOI:** 10.1371/journal.pone.0294958

**Published:** 2023-11-27

**Authors:** Yu-Liang Zhang, Jin-Fu Li, You-Qu Zheng

**Affiliations:** 1 College of Mechanical Engineering & Key Laboratory of Air-driven Equipment Technology of Zhejiang Province, Quzhou University, Quzhou, China; 2 College of Mechanical Engineering, Zhejiang University of Technology, Hangzhou, China; NED University of Engineering and Technology, PAKISTAN

## Abstract

The study on transient characteristics of pump as turbines during atypical startup has not been deeply explored yet. In order to reveal the transient characteristics of a small centrifugal pump reversing as turbine during startup process in this paper, the transient hydraulic performance experiments are conducted for three steady rotational speed cases. Under the condition of each case, three, five and, three valve opening scenarios are completed to measure the performance. The dimensionless analysis are also employed so as to better reveal transient behavior of the pump as turbine during atypical startup. The results show that the rise rate of each performance parameter is different, wherein the shaft power and rotational speed have the fastest rising rate, followed by the flow rate, and the head rise is the slowest. It is clearly seen that the shock phenomenon in static pressure easily occurs at the outlet of pump as turbine. With the increase of valve opening, the dimensionless flow rate, head and, power coefficient all show the evolution trend of gradually increasing.

## 1 Introduction

In the petrochemical, refining, and desalination industries, a large amount of high-pressure fluid is depressurized to low pressure or discharged directly through pressure-reducing valves. In seawater desalination systems, the pressure of waste-concentrated brine discharged from membrane vessels is as high as 5.5 to 6.0 MPa [1]. If it is discharged directly without energy recovery, it will cause serious energy waste. At present, centrifugal pump reversal as a hydraulic turbine (pump as turbine, abbreviated PAT) is an essential means to recover liquid pressure energy.

It is found from the published literature that most scholars obtain the hydraulic performance of PATs based on their hydraulic performance under pump operating conditions. Williams et al. conducted forward and reverse performance comparison experiments on 35 pumps to compare the accuracy of eight prediction methods [2]. It was found that all eight prediction methods were partially accurate. Singh et al. proposed a unified prediction model to describe the performance of PATs based on the primary flow theory of pumps [3]. The model can display the prediction error, wherein the specific speed is considered a variable. Miao et al. has presented a radial surface optimization design method for the pump as turbine impeller, and the optimized pump as turbine efficiency was improved by 2.28% at the optimal service point. [4]. Wang et al. derived a prediction equation for the performance of turbine efficiency point based on pump and turbine efficiency with turbine inlet slip by analyzing the impeller’s inlet and outlet velocity triangle and compared six pumps as turbine with revolutions of 9.0–54.8 for experimental and numerical simulations. The results showed that the slip coefficient of the pump condition is greater than that of the turbine condition at the design condition point [5]. Huang et al. theoretically derived the performance equation of turbine impeller based on the effect of vane outlet slip and finite vane number. They proposed a prediction equation for the optimal operating point of PAT, which pointed out the matching range of optimal parameters of PAT and external load [6]. The essence of this study is to apply the area ratio principle of a pump to the optimal design of the PAT, which has a good practical value.

Derakhshan et al. theoretically derived a method for calculating the optimal efficiency point of PATs. The results confirmed that the predicted value based on this method is slightly lower than the experimental value [7]. Shi et al. proposed three methods for calculating the slip coefficient of the impeller outlet of PAT based on different assumptions [8]. It was found that the ratio of the induced velocity on the blade pressure side over the slip velocity on the impeller outlet was equal to the ratio of the areas of two triangles with the pressure side and impeller outlet as one curved edge, respectively, the slip factor is subject to excellent accuracy. This literature can be beneficial to improve the prediction accuracy of the theoretical performance of PAT. Li conducted a detailed theoretical study on the relationship between the performance curves of the pump and PAT when conveying viscous oil and proposed multiple expressions for correction factors describing the performance correlation between them [9]. This literature provides an essential reference for selecting PAT products with viscous oil as the working medium. The delivering medium in the study is a high-viscosity viscous oil, which is different from the previous clear water medium. Abazariyan et al. studied the effect of viscosity on the pump as a turbine and proposed a correlation between the calculated efficiency and the flow coefficient and Reynolds number [10]. Tan et al. proposed a performance prediction method with high accuracy [11]. The specific speed and impeller diameter characteristics are approximately linear between the pump operating conditions and the turbine operating conditions. Lin et al. proposed a theoretical method for predicting the best efficiency point of PAT based on the impeller-volute matching principle, and the experimental results of the head and discharge conversion factors and the predicted results obtained through theoretical or statistical methods were compared to verify the effectiveness of the method proposed [12]. Ye et al. numerically simulated a multistage pump as a turbine and studied its flow characteristics using entropy production theory. The results show that: the numerical simulation results are basically consistent with the experimental results, and the guide vane, impeller, inlet and outlet volute, front and back chambers, and balance disk are the primary sources of energy loss in multistage PAT, and the higher relative velocity is accompanied by larger energy loss [13]. Chen et al. investigated the effects of thermodynamic cavitation and tip leakage vortex (TLV) on hydraulic losses in the liquid Cryogenic cavitation was investigated to determine the effect of heat transfer on energy dissipation in a liquid nitrogen inducer [14].

Wang et al. designed four forward-curved impellers with different blade inlet and outlet angles and found that the efficiency curve of the forward-curved impeller became flatter, and the optimal efficiency increased significantly compared with the backward-curved impeller [15]. Doshi et al. chamfered the inner side of the hub and shroud [16]. Moreover, the blade inlet of the PAT impeller is also chamfered. The hydraulic loss could be effectively reduced, and the conveying efficiency could be improved. What’s more, modifying the backward-curved blade angle could cause hydraulic loss to reduce to 5–10%. Li et al. found that the flow rate and the energy head consumed under the optimal working condition of the turbine increased with the increase of viscosity. At the same time, the hydraulic efficiency showed a decreasing trend, and the output shaft power was mainly determined by the liquid density [17,18]. It is also pointed out that viscosity more severely impacts turbine performance than pumps. The net positive suction head (NPSH) of the PAT increases with the increase of liquid viscosity, but the magnitude is relatively small. Hu et al. [19] investigated the hydraulic characteristics of pumps as turbines under transient flow conditions. It was shown that the transient flow conditions significantly affect the efficiency of the pump as a turbine. With increasing flow rate, the hydrodynamic force on the impeller and pressure fluctuations on the volute first decrease and then increase, reaching a minimum value near the design flow rate. Du et al. investigated the internal flow behavior of a centrifugal pump operating as a turbine at different speeds and flow rates and analyzed the vortex behavior, turbulent kinetic energy, pressure, and velocity distribution [20]. The results showed that vortex and turbulent kinetic energy affected the internal flow at PAT, with flow instability and local flow structure at higher speeds and flow rates. Vortex and turbulent kinetic energy increased with increasing speed. The pressure and velocity distribution is higher at the inlet than at the outlet. Adu et al. used the k-e turbulence model to simulate the pump as turbine operation for different diverter vane positions and compared it with the experimental results [21]. The simulation results were in excellent agreement with the experimental results, and with the diverter, vanes are more suitable for the pump as a turbine operating. Zhang et al. measured the transient hydraulic performance of a centrifugal pump as turbine during an atypical startup period [22]. The result showed that the impact phenomena occur in both flowrate curve and outlet static pressure curve in an atypical startup.

CFD (computational fluid dynamics) has become one of the important methods for studying PATs [23–25]. The authors’ preliminary numerical calculations found that as the liquid viscosity increased, the morphology and size of the lobe cavities at the outlet side of the vane under each operating condition did not change significantly, but the length of the cavity at the tip of the drain cone gradually became shorter [18].

To sum up, it can be found that scholars have carried out lots of studies to predict the hydraulic performance of PAT from various aspects. However, these studies aim for stable working conditions, i.e., the performance prediction study under the steady rotational speed condition. In contrast, the study on starting characteristics of the PAT has rarely been involved. In this paper, the performance experiments will be conducted on the external characteristics of a small centrifugal pump reversing as a turbine during an atypical startup process, and the transient characteristics of the PAT during this atypical startup process are further revealed with the help of dimensionless analysis.

## 2 Test rig and physical model

### 2.1 Test rig

**Fig 1:**
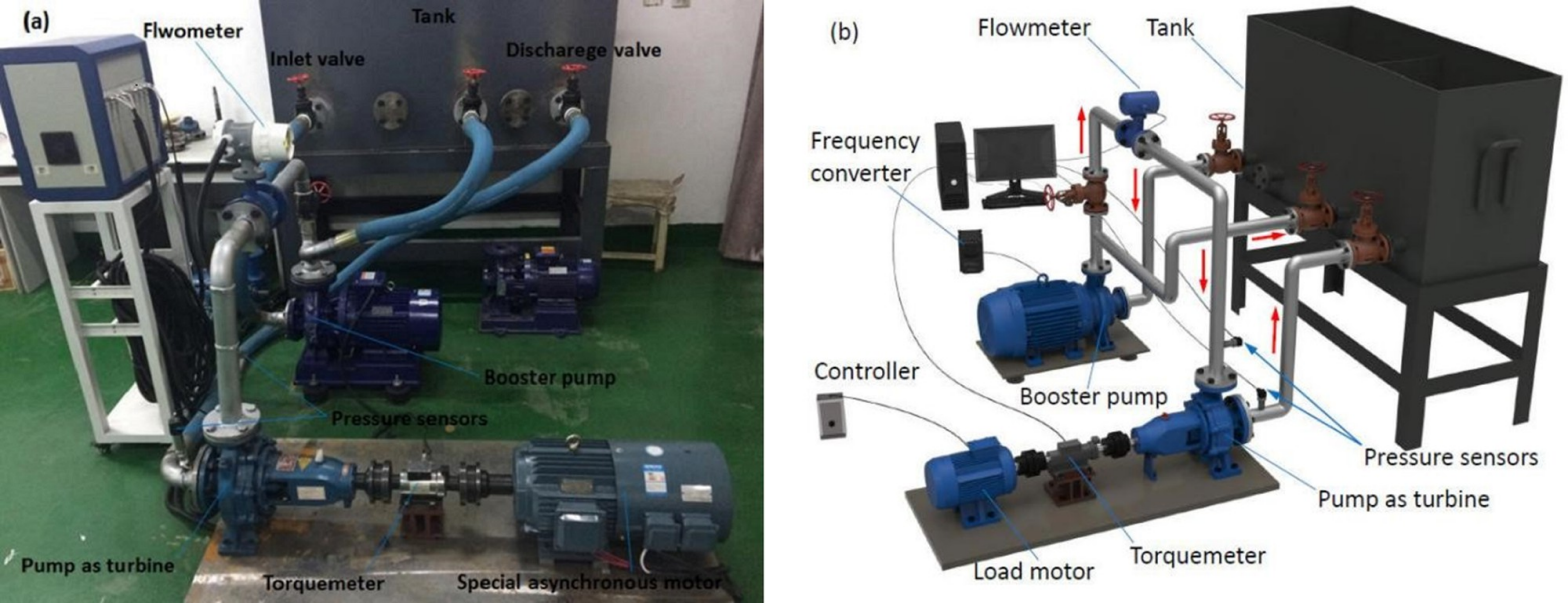
Test rig (a) physical (b) model.

The test rig of PAT in this paper is shown in Fig 1. It is an open test rig. The test rig mainly comprises a booster pump unit, PAT unit, tank, piping system, and test system, etc. The drive source of the booster pump is a three-phase asynchronous motor, and its rotational speed is controlled by a frequency converter. The PAT unit mainly consists of PAT, a dynamic torque sensor, and a special three-phase asynchronous motor for frequency control. The dynamic torque sensor is an NH-901 type strain gauge torque sensor with a range of 0–100 N m and a measurement accuracy of ±0.5% F.S, it is a sampling frequency of 1000 times per second. The rotational speed is measured by a photoelectric encoder mounted on a dynamic torque sensor with a range of 0–9999 rpm, a measurement accuracy of ±1 rpm, and a sampling frequency of 1000 times per second. The particular three-phase induction motor is used to absorb the shaft power generated by the PAT. The testing system mainly includes an electromagnetic flowmeter, two inlet and outlet pressure sensors, and a rotational speed torque sensor. The model of the electromagnetic flowmeter is ZHLDG-40, the range is 0~70.65 m^3^/h, the measurement accuracy is ±0.5% F.S, and the sampling frequency is 1000 times per second. Both the inlet and outlet pressure sensors are WIKA type, and their ranges are from 0 to 1.6 MPa and from −0.1 to 0.5 MPa, respectively. The measurement accuracy is ±0.5% F.S, and the sampling frequency is 1000 times per second. As a test rig for experimental measurement, the main purpose is to investigate the external and transient characteristics of the pump as turbine during atypical startup. And, the efficiencies of the pump and pump as turbine, which are the most important components of the test rig, are shown as follows: the booster pump ISW-65-160 is 63%, and the pump as turbine of the prototype pump IS-80-50-200 is 59%.

### 2.2 Physical model

**Fig 2:**
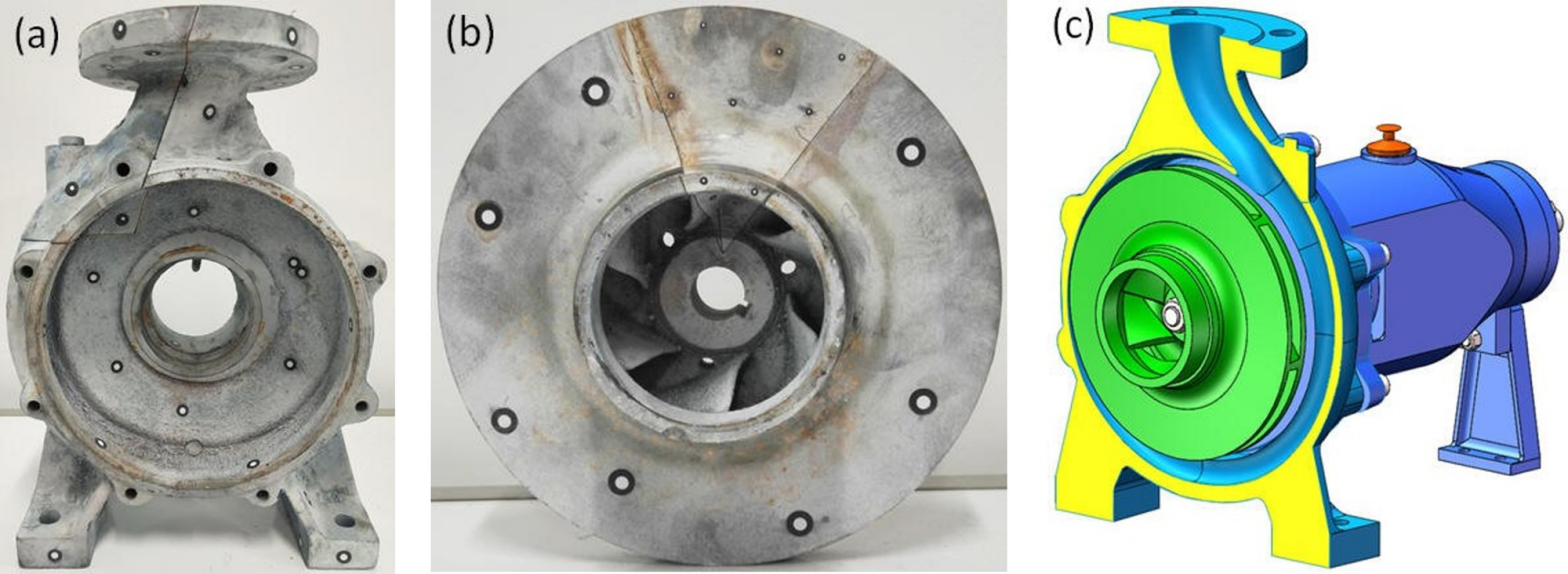
PAT (a) volute (b) impeller (c) assembly model.

A direct-connected pipeline centrifugal pump is selected as the booster pump; its type is ISW-65-160(1). The parameters at the best efficiency point, namely the rated parameters, are flow rate (*Q*_r_) 50 m^3^/h, head (*H*_r_) 32 m, and rotational speed (*n*_r_) 2900 r/min. The prototype centrifugal pump reversing for turbine is a centrifugal water pump, as shown in Fig 2, the type is IS80-50-200J. Likewise, t he parameters at its best efficiency point, namely the rated parameters, are flow rate (*Q*_r_) 25 m^3^/h, head (*H*_r_) 12.5 m, and rotational speed (*n*_r_) 1450 r/min. The diameters of the pump inlet and outlet are 80 mm and 50 mm, respectively. Six twisted blades are used. The inlet blade angle of the flow line in the middle of the blade is 20° and the outlet angle is 42.5°. The blade is 3.5mm thick, the inlet diameter of the blade is 66 mm, and the outer diameter of the impeller is 202 mm. The impeller inlet is 34 mm wide and the outlet is 9.0 mm wide. The inlet width of the spiral volute is 24 mm, and the diameter of base circle of the volute is 210 mm.

### 2.3 Experimental methods and scheme

During an atypical startup process, the testing steps of the transient performance of the pump as turbine are as follows. Before testing, the entire circulating piping system is filled with water, and the free liquid surface of the water tank is consistently higher than that of the booster pump, the pump as turbine, and the entire pipe system. A 50 Hz current output frequency is set at the frequency converter (the steady rotational speed of the booster pump that corresponds to 220 V voltage is 2900 rpm). And starting the booster pump unit. When the rotational speed stabilizes to approximately 2900 rpm, adjust the opening of the discharge valve and the load of the three-phase asynchronous motor for variable frequency speed regulation repeatedly. When both the flow rate and rotational speed of pump as a turbine reach to expect values, keep the valve opening and the control load constant. Finally, the working state of the pump as turbine at a certain steady rotational speed and steady flow rate is obtained. Then, the booster pump unit is shut down until the water in the entire circulating pipe system, including the water tank, is at a standstill. Next, starting the booster pump unit. At this time, the pressure sensor will record the instantaneous static pressure value at the inlet and outlet of the pump as turbine in real time, the electromagnetic flowmeter will record the instantaneous flow rate value passing through the pump as turbine in real time, and the torque meter will record the instantaneous rotational speed and torque of the pump as turbine in real time, so that. Thus, the instantaneous hydraulic performance of the pump as turbine is obtained.

The testing process is as follows. Open each valve, start the booster pump, and pressurize the fluid from the water tank. Part of the pressurized water flows back to the water tank, and another part flows into the pump as turbine. After the pump as turbine operates, the water flows back to the water tank. The impeller of the pump as turbine begins to rotate after being worked by the pressurized water flow, and it drives the torquemeter and the three-phase asynchronous motor for variable frequency speed regulation to rotate. This starting mode is different from the general mode, called the atypical startup.

In this paper, the experiments on different valve openings under the stable speed of 400 r/min are firstly conducted, then the stable speed of the PAT increases to 600 r/min, and finally to 800 r/min, which are called as low, medium, and high speed, respectively. The corresponding starting process is also called low-speed start, medium-speed start, and high-speed start, respectively. The detailed testing scheme is shown in Table 1.

**Table 1:** Testing scheme.

startup status	targeted stable speed *n*_f_(r/min)	targeted stable flowrate *Q*_f_(m^3^/h)					
		20	25	30	35	40	45
low-speed start	400	✓		✓		✓	
medium-speed start	600		✓	✓	✓	✓	✓
high-speed start	800		✓		✓		✓

## 3 Results analysis

### 3.1 Steady performance

**Fig 3:**
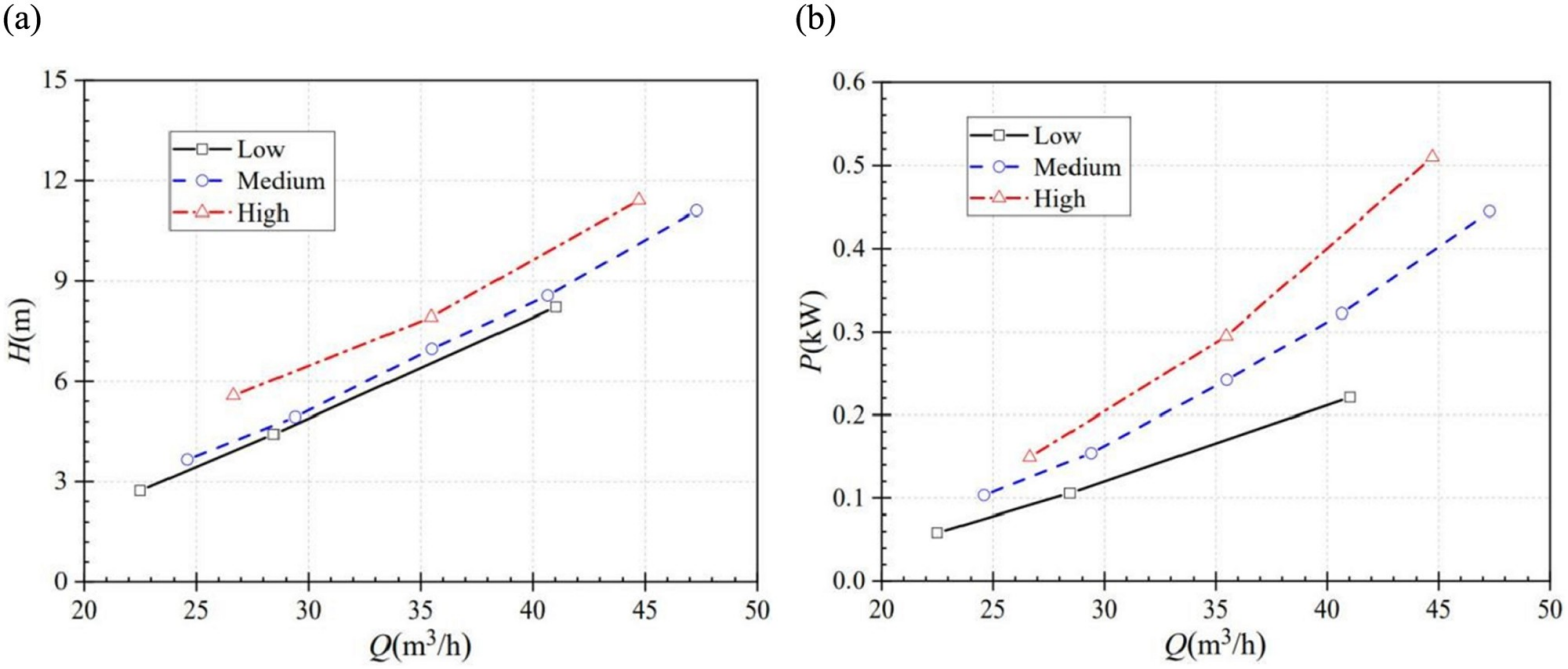
Steady performance curves (a) head-flow curve (b) power-flow curve.

The steady head-flow curves are shown in Fig 3 at three steady rotational speeds. It is obvious that both the head and the power increase with the increase in flow rate. The difference among the three power curves would be more significant with the flow rate increase. This change tendency is consistent with the existing literature [19,26].

### 3.2 Low-speed start

**Fig 4:**
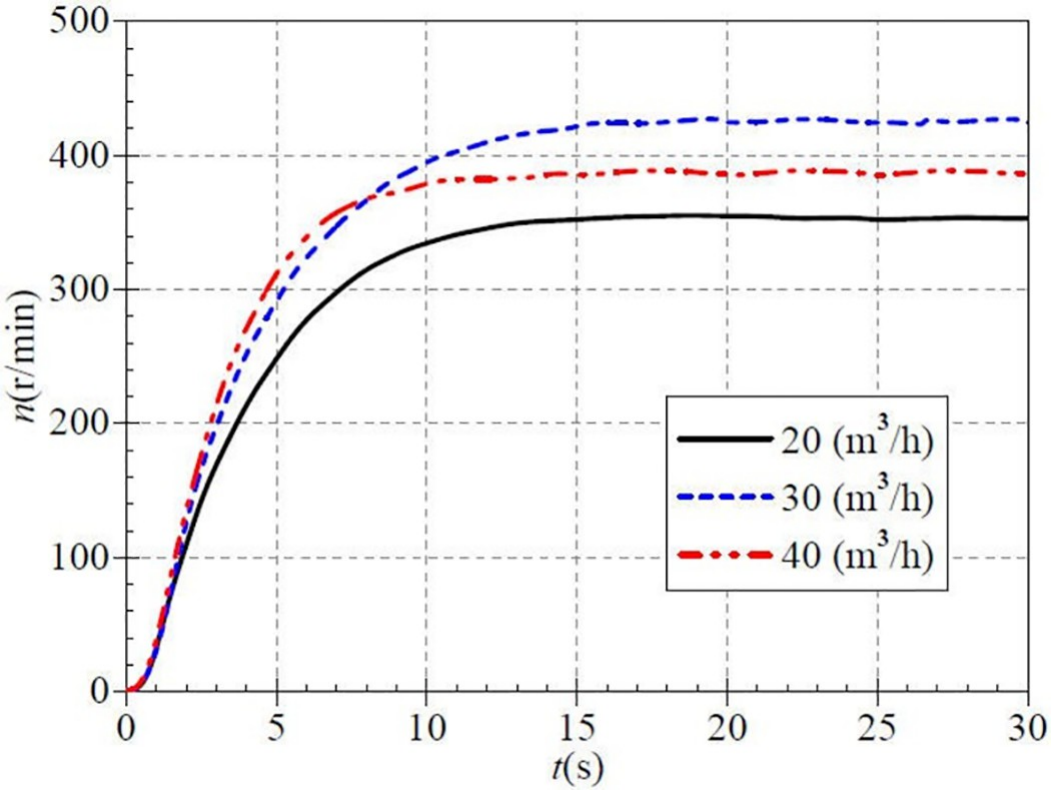
Rotational speed rise (low-speed start).

The stable flow rate after startup is controlled by adjusting the opening of the outlet valve. The speed rise curve during low-speed atypical startup for the three valve openings is shown in Fig 4. Three stable flow rates are 20 m^3^/h, 30 m^3^/h, and 40 m^3^/h, which corresponded to small, medium, and large valve openings. Obviously, the speed curves under different valve openings have similar evolutionary characteristics: a slow rise, then a rapid rise, and then a slow rise to the final stable value.

The rotational speed rise is extremely slow before 1.20 s. This is because the rotating parts of PAT are stationary at the beginning of the startup process; the torque generated by the pressurized fluid first overcomes the frictional resistance of the rotating parts. After overcoming the frictional resistance, the rotational speed begins to rise rapidly. Among them, the rising rate of rotational speed is significantly higher in the medium and large opening cases than in the small opening case. The main reason for this phenomenon is that because the rotational speed rises more rapidly, the torque generated by the larger flow rate is also greater.

At about 8.1s, the rotational speed in the medium opening case exceeds the speed in the large opening case for the first time. It completely exceeds the other two opening cases after that. At about 16.0s, the rotational speed almost stops rising and reaches a stable value. The stabilization values for the small, medium, and large openings are about 353.3 r/min, 425.5 r/min, and 388.5 r/min, respectively, and the stabilization rotational speed value for the medium opening case (i.e., the stable flow rate of 30 m^3^/h) is significantly higher than the other two cases. The difference between the experimental and targeted speeds for the three valve opening cases may be related to the unstable voltage in the testing process.

**Fig 5:**
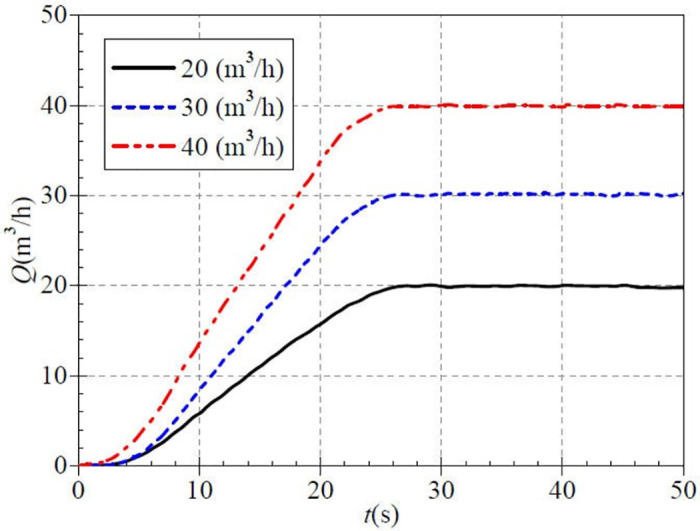
Transient flow rate rise (low-speed start).

The instantaneous flow curves measured during the low-speed atypical startup are shown in Fig 5. The measured steady flow values for this paper’s three valve opening scenarios were 19.989 m^3^/h, 30.176 m^3^/h, and 39.881 m^3^/h. The differences from the targeted values of 20.0 m^3^/h, 30.0 m^3^/h, and 40.0 m^3^/h are very slight. This is because the stable working flow rate after startup is achieved by adjusting the outlet valve opening in present experiments, which is a dynamic adjustment, so the measured values do not differ much from the targeted solutions. Also, in the atypical startup process, the three flow rate curves generally show similar evolutionary characteristics, all characterized by a slow rise, then a rapid rise, and then a slow rise to a steady flow. The measured flow rate are 0.363 m^3^/h, 0.364 m^3^/h, and 1.545 m^3^/h for the small, medium, and large open cases before about 3.5 s. The time required for the three flow rate curves to rise to their respective stable values is about 27.0 s, 26.5 s, and 27.0 s, respectively. The flow rate rises lag behind the rotational speed rise. The three flow rate curves still have extremely slight fluctuations after reaching stability due to the rotor-stator interaction of PAT, and its analysis will not cause a significant impact.

**Fig 6:**
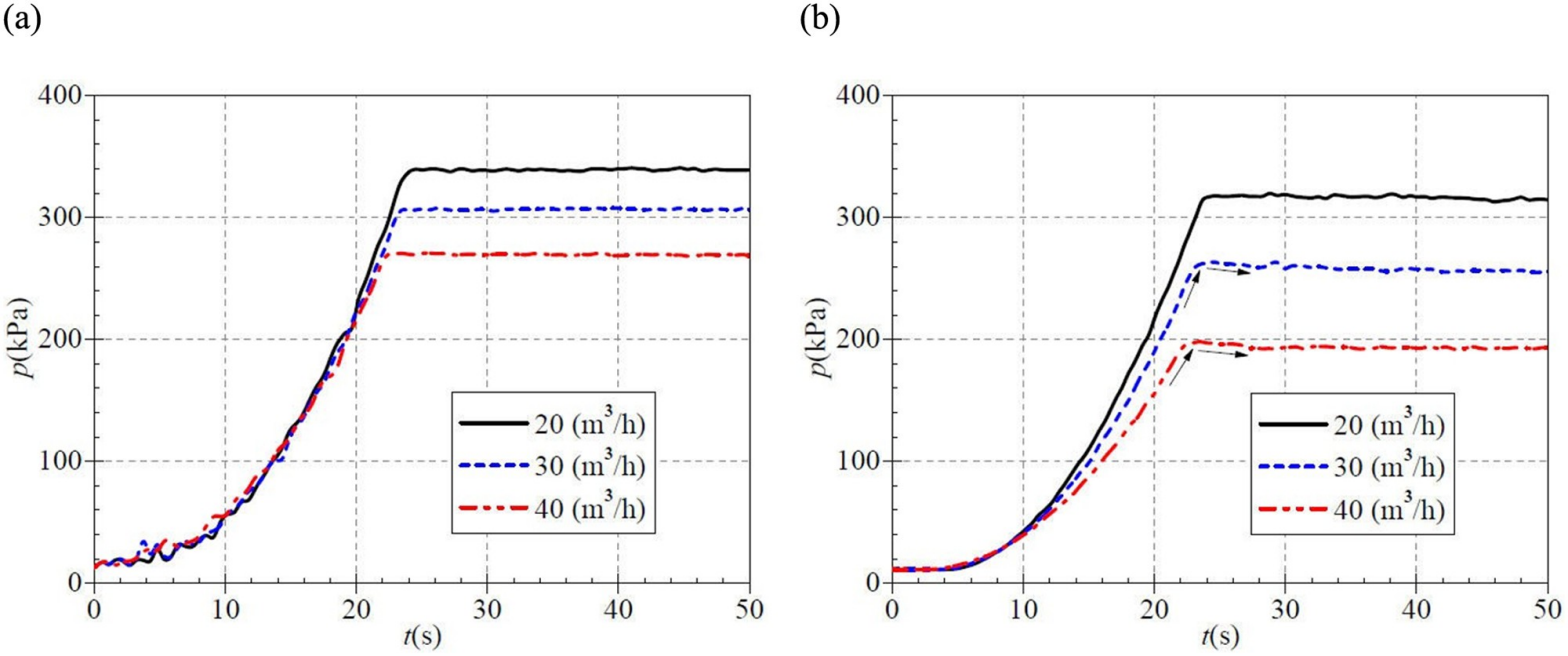
Transient static pressure rise (low-speed start) at (a) inlet (b) outlet.

Under the low-speed startup situation, the transient static pressure curves measured at the inlet and outlet of the PAT are shown in Fig 6. It is seen from Fig 6(a) that the static pressures at the initial moment of atypical startup are about 13.0 kPa. With the rapid increase of the rotational speed of the booster pump, the static pressure at the inlet of the PAT also shows a rising trend. The rise of the static pressure at the PAT inlet is the same before 20.0 s under the condition of three valve openings. The differences among them are minor. After 20.0 s, with the increase of valve opening of PAT, the rising rate of the inlet static pressure decreases, and the pressure rises slowly. In the case of small, medium, and large valve openings, the stable static pressures at PAT inlet after startup are about 339.636 kPa, 306.594 kPa, and 270.088 kPa, respectively, and the corresponding rise times are about 24.6 s, 23.6 s, and 22.5 s. As the valve opening increases, the time required for the static inlet pressure to rise to a stable value shows a slight tendency to advance during atypical startup.

It has been found in the literature that there is a sudden increase and then a decrease in the flow rate and pressure parameters during the pump startup process, and this phenomenon was been defined as "shock".[27] Fig 6(b) shows that in the case of small, medium, and large three valve openings, the outlet static pressure curves of PAT as a whole show a slow rise, then a rapid rise, and then down to a stable value, i.e., the static outlet pressure of PAT generally has the shocked phenomenon during the atypical startup process. It can be found that the outlet static pressure rises extremely slowly before 5.0 s, and then the rising rate is gradually accelerated. The maximum instantaneous static pressure values are 263.572 kPa and 198.176 kPa for the medium and large valve openings, corresponding to about 24.5 s and 23.4 s. It can be found that the larger the valve opening, the earlier the shocking phenomenon of outlet static pressure occurs. The final stable static pressures after atypical startup are about 317.828 kPa, 258.964 kPa, and 192. 806 kPa, respectively. The shock static pressures (the difference between the maximum value and the stable value) are 4.086 kPa and 5.370 kPa for the two valve opening cases of medium and large, respectively. This indicates that the shocking phenomenon of outlet static pressure is the most intense in the case of the large valve opening.

**Fig 7:**
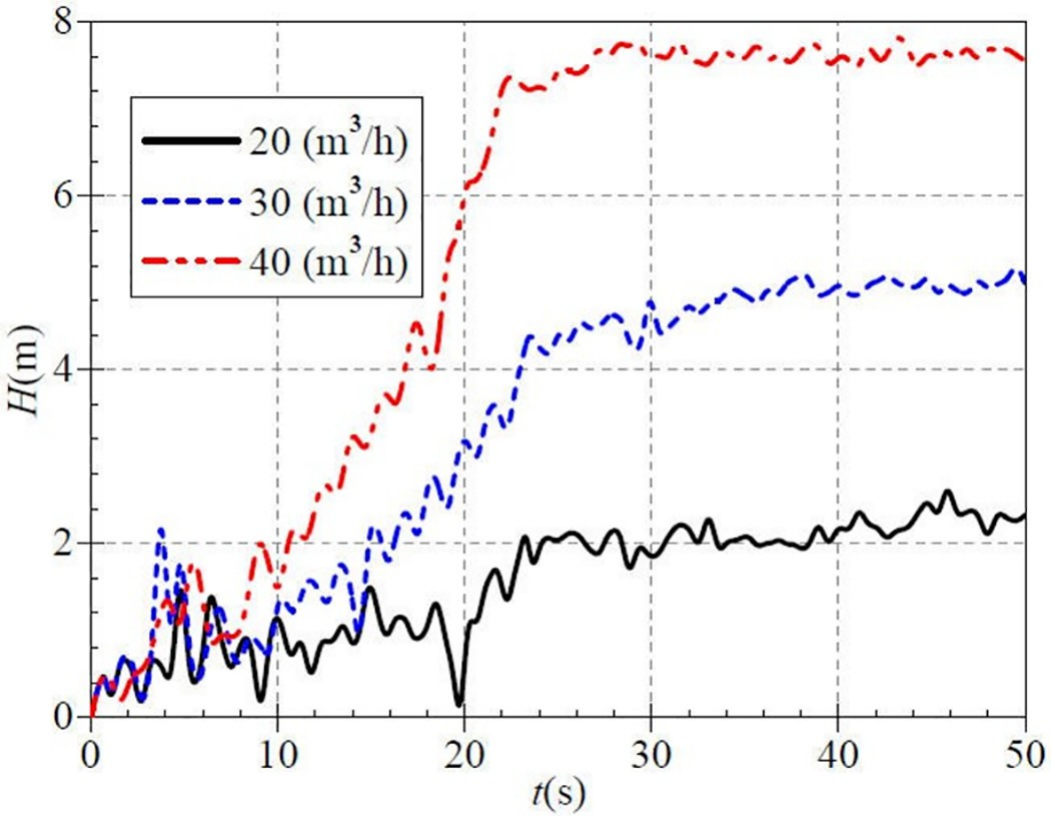
Transient head rise (low-speed start).

Although Fig 6 has shown the variation of pressure at the inlet and outlet of the pump as turbine during start-up, but the work capacity of the pump as turbine cannot be well represented. As an important performance parameter, head can indicate the net increase in energy per unit mass of fluid passing through the pump. Therefore, it is necessary to investigate the head characteristics during pump as turbine start-up. Fig 7 shows that the evolution curve of the instantaneous head fluctuates very sharply. In the case of a small valve opening, there are two local maxima and two local minima in the rising stage of the head curve. One local maximum and two local minima are in the corresponding head curve for medium valve opening. For the large valve opening, there is one local minimum. It can be seen that compared with the two valve opening cases of medium and large, the head rise curve in the small valve opening case fluctuates most dramatically.

The average heads after startup are 2.29 m, 4.92 m, and 7.62 m for the small, medium, and large valve opening cases, respectively. The instantaneous head curves do not rise after about 28.0 s, after which each head curve fluctuates within a specific range. With the increase of outlet valve opening of PAT, the head showed an increasing trend.

**Fig 8:**
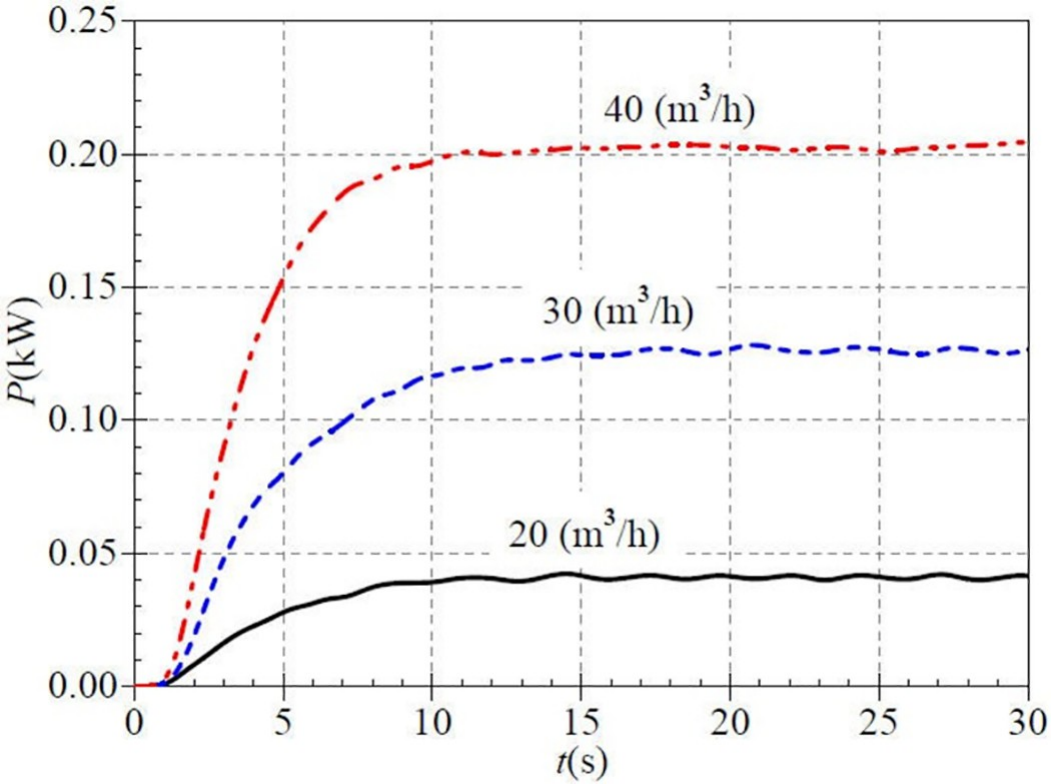
Transient shaft power rise (low-speed start).

Fig 8 shows that the overall shaft power curves show an evolutionary process of slow rise, then fast rise, and slowly rise again at all three valve openings, i.e., there are two slow and one fast rising evolutionary feature. The shaft power rises extremely slowly before 1.0 s. The rising rate differs for each valve opening case in the rapid rise phase. At 5.0 s, the corresponding instantaneous shaft powers are 0.028 kW, 0.080 kW, and 0.153 kW for small, medium, and large valve openings, respectively, and the ratios of transient shaft power to their final steady values are about 0.667, 0.630, and 0.754, respectively. The shaft power rises the fastest in the large valve opening case, followed by the small valve opening, and the slowest is the medium valve opening. Then the shaft power curve enters the slow growth stage and reaches its stable values at about 14.5 s, 17.7 s, and 16.6 s, corresponding to the stable values of 0.042 kW, 0.127 kW, and 0.203 kW, respectively, which fully shows that as the valve opening increases, the shaft power of PAT is also more significant.

### 3.3 medium-speed Start

**Fig 9:**
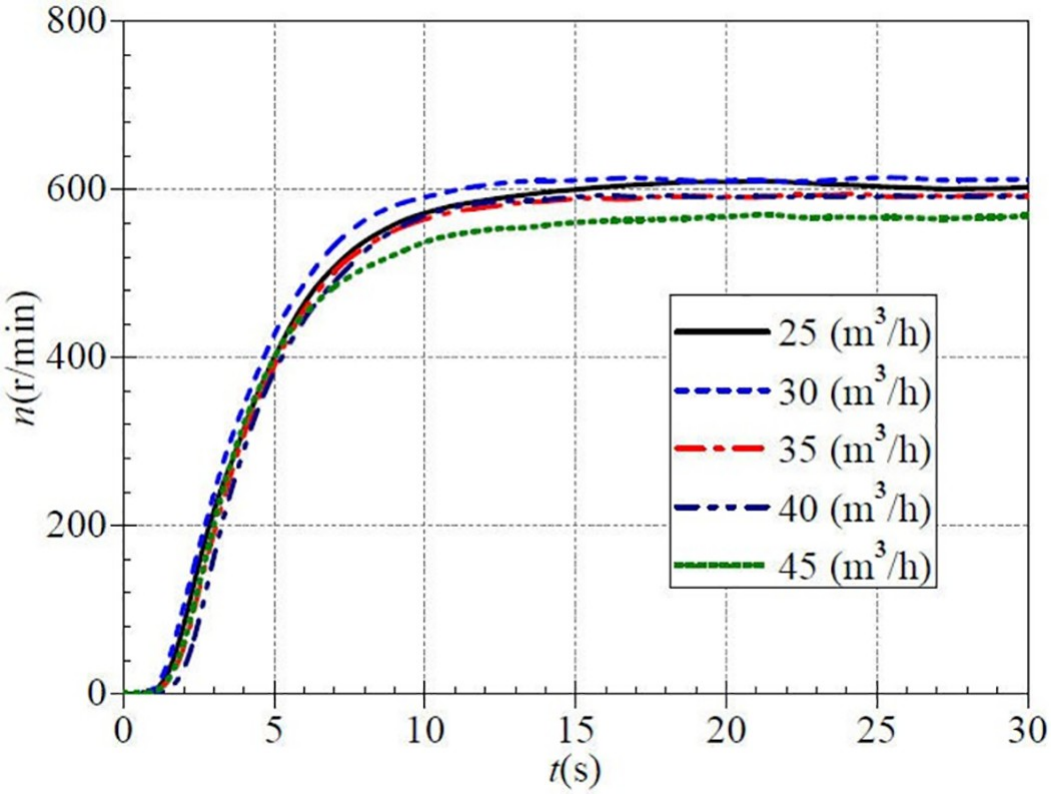
Rotational speed rise (medium-speed start).

Fig 9 shows the measured rotational speed curves during the medium-speed startup process. The stable flow values are respectively adjusted as 25 m^3^/h, 30 m^3^/h, 35 m^3^/h, 40 m^3^/h, and 45 m^3^/h in this case, which corresponds to the five valve openings of small, lower middle, middle, upper middle, and large, respectively. The five rotational speed rise curves have a similar evolutionary history, which shows a slow rise, then a rapid rise, and then a slow rise to the final stable value.

The rotational speed rise is prolonged until about 1.0 s. The rising rate of instantaneous rotational speed in the lower middle valve case (30 m^3^/h) is faster than in the other four cases. After 1.0 s, the rotational speed enters a rapid increase phase. In this phase, the rising rate is more rapid in the lower medium valve opening. Consistent with the low-speed startup process, the instantaneous speed at the small and medium valve opening (i.e., the steady flow rate of 30 m^3^/h) is significantly larger than the other four valve openings, while the instantaneous speed at the upper middle valve opening is the smallest among the five valve openings. At about 7.0 s, the speed rise becomes slow again, and the instantaneous speed in the case of the upper middle valve opening exceeds that of the large valve opening. The speed values in the large valve opening are the minimum after that. After about 16.0 s, the rotational speed rises gradually to the stable values, which are about 609.132 r/min, 613.971 r/min, 592.316 r/min, 593.121 r/min, and 570.300 r/min, respectively. The steady speed achieved at each valve opening differs from the targeted rotational speed. Moreover, at a steady flow rate of 30 m^3^/h, the corresponding speed rise is the fastest and has the highest stable speed value.

**Fig 10:**
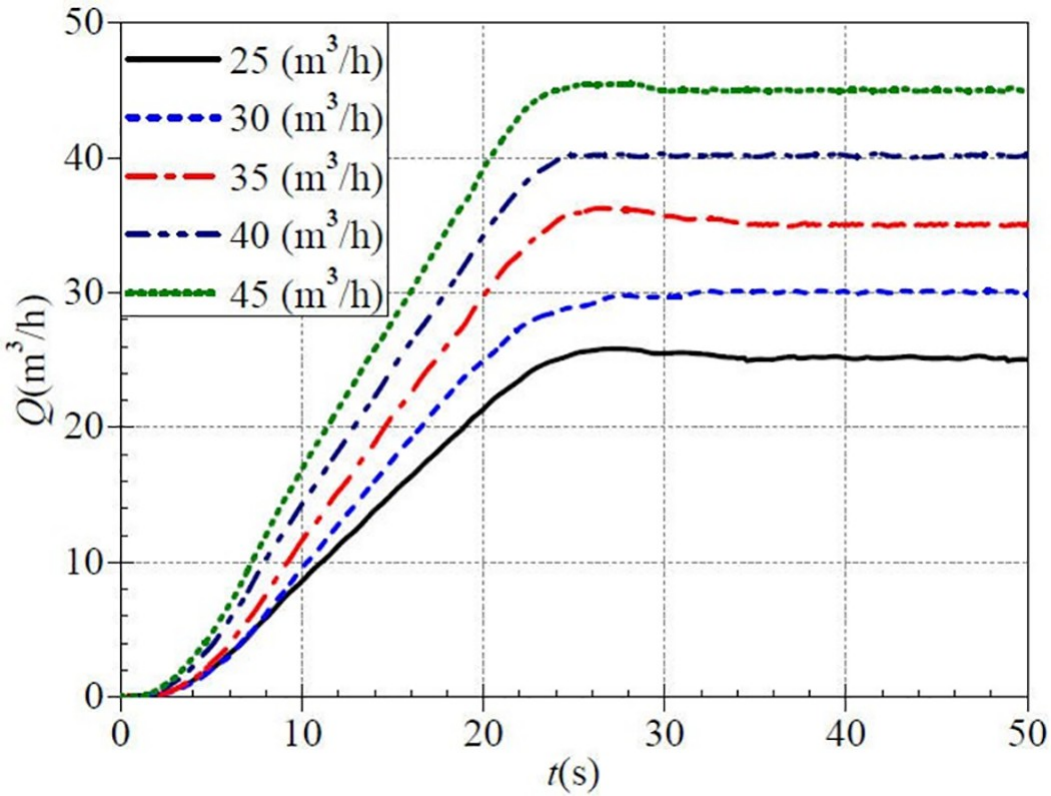
Transient flow rate rise (medium-speed start).

The instantaneously measured flow rate curves during the medium-speed startup are shown in Fig 10. Again, since the flow rate regulation is an active control, the five measured steady flow rates are 24.966 m^3^/h, 30.084 m^3^/h, 35.038 m^3^/h, 40.197 m^3^/h, and 44.968 m^3^/h, and the difference from the targeted values is very small. All flow rate curves show an evolutionary characteristic of slowly increasing, rapidly rising to a maximum value, and slowly decreasing to a stable value. There is no apparent correspondence between the valve opening and the rising rate of flow rate at the medium-speed start compared to the low-speed start. In the five valve opening cases, except for the two flow rate curves of 30.0 m^3^/h, and 40.0 m^3^/h, the other three flow rate curves rise rapidly to a maximum value and then fall slowly until they drop to their respective stable values. Unlike the low-speed start, there is a flow rate shock phenomenon during the medium-speed start, with the maximum values of 25.846 m^3^/h, 36.273 m^3^/h, and 45.488 m^3^/h for small, medium, and large valve openings, respectively, and the ratios of the maximum values to the final stable values are 1.035, 1.035 and 1.012 after startup. In the case of three valve openings, the shock flow rates (defined as the difference between the maximum value and the stable value) are 0.880 m^3^/h, 1.232 m^3^/h, and 0.520 m^3^/h, respectively. This indicates that the flow rate shock phenomenon is the strongest in the middle valve opening case and relatively insignificant in the rest of the valve opening cases.

**Fig 11:**
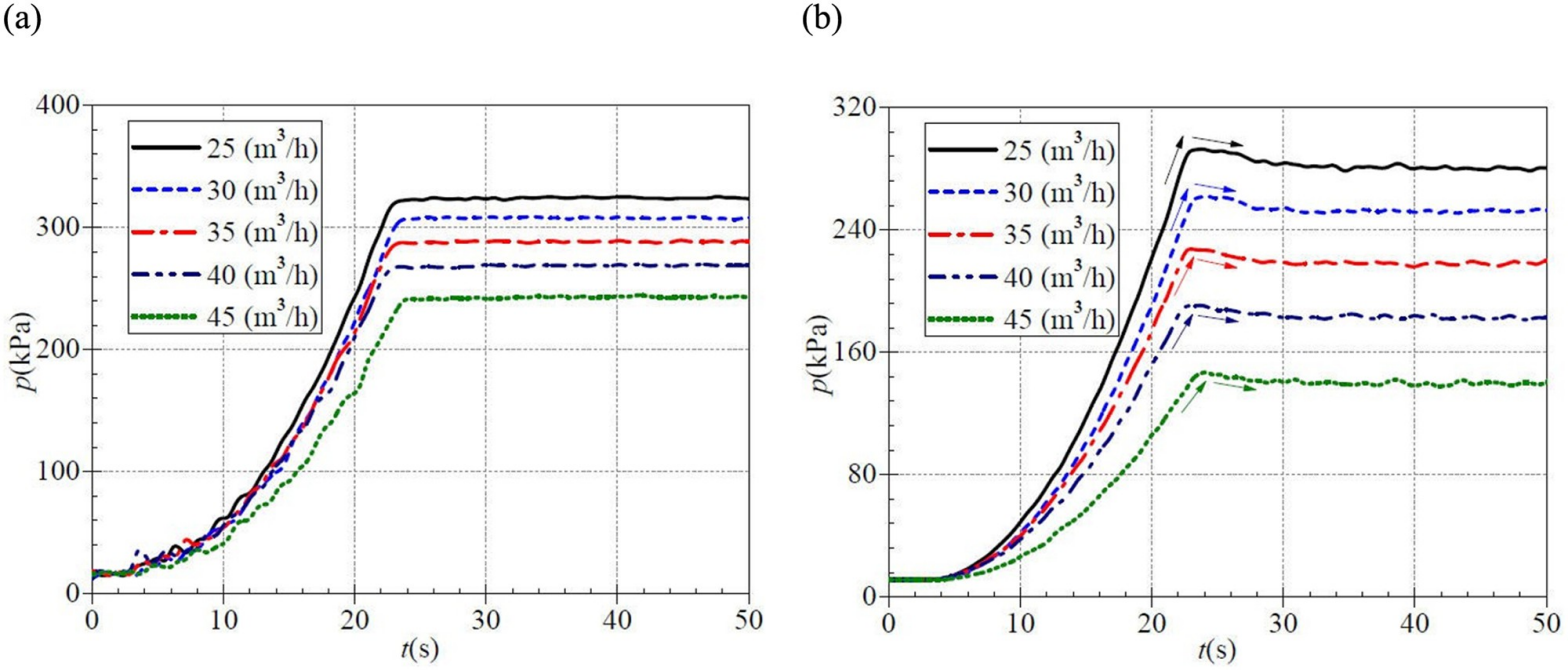
Transient static pressure rise (medium-speed start) at (a) inlet (b) outlet.

Fig 11(a) shows that at the beginning of the startup period, each static pressure curve rises slowly, displaying a fluctuating upward trend. After that, the inlet static pressure of the PAT shows rapid growth. For five valve openings, the stable inlet static pressure after startup are 323.888 kPa, 307.197 kPa, 287.696 kPa, 269.576 kPa, and 242.806 kPa, corresponding to a rise time of 24.7 s, 25.6 s, 25.7 s, 25.6 s, and 25.9 s. Unlike the low-speed startup, the time required for the inlet static pressure of PAT to rise to the steady value during medium-speed shows a slight delay trend with the increase of valve opening. In addition, the stable inlet static pressure decreases as the steady flow rate increases.

Fig 11(b) shows that the outlet static pressure of PAT as a whole shows a slow rise, then a rapid rise. Then down to a stable value of the evolution characteristics, the outlet static pressure of PAT in the atypical startup process has generally shock phenomenon. The outlet static pressure rises very slowly before 6.0 s, and the rising rate is gradually accelerated. In the five valve opening cases, the maximum instantaneous static pressures are 292.784 kPa, 262.256 kPa, 227.502 kPa, 190.527 kPa, and 146.911 kPa, and the corresponding moments are about 23.8 s, 24.1 s, 23.0 s, 23.0 s, and 24.1 s. It can be found that the time of shock static pressure did not change with the change of the valve opening; that is, the shock phenomena of outlet static pressure and the valve opening is unrelated. The stable outlet static pressures are about 281.482 kPa, 254.021 kPa, 218.745 kPa, 182.744 kPa, and 140.104 kPa after startup, and the shock static pressure (the difference between the maximum value and the stable value) are 11.302 kPa, 8.235 kPa, 8.757 kPa, 7.783 kPa, and 6.807 kPa. In the process of medium-speed start, the shocking phenomenon of static pressure is the most intense in the case of a small valve opening, and it also shows a trend of weakening with the increase of valve opening. In addition, the outlet static pressure also indicates a certain degree of fluctuation due to rotor-stator interaction.

**Fig 12:**
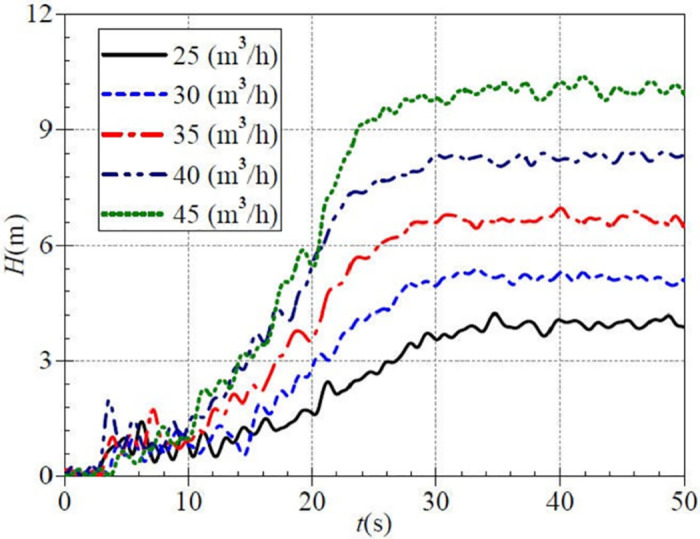
Transient head rise (medium-speed start).

Fig 12 shows that the evolution curve of the instantaneous head fluctuates sharply, especially before 10.0 s. This phase has several extreme points, and there is no clear rule for the instantaneous head under each valve opening. Taking the upper middle valve opening as an example, the instantaneous heads reach local extreme values of 1.93 m, 0.74 m, 1.47 m, and 0.64 m at 3.5 s, 4.6 s, 5.7 s, and 7.6s. And then, the instantaneous head curve rises to a stable value. In addition, the final average heads after startup are 3.94 m, 5.17 m, 6.68 m, 8.30 m, and 10.04 m for the five valve openings, and there are still small fluctuations after the rising to the stable value. It can be found that the head fluctuation is reduced, and the stable head is increased compared with the low-speed start.

**Fig 13:**
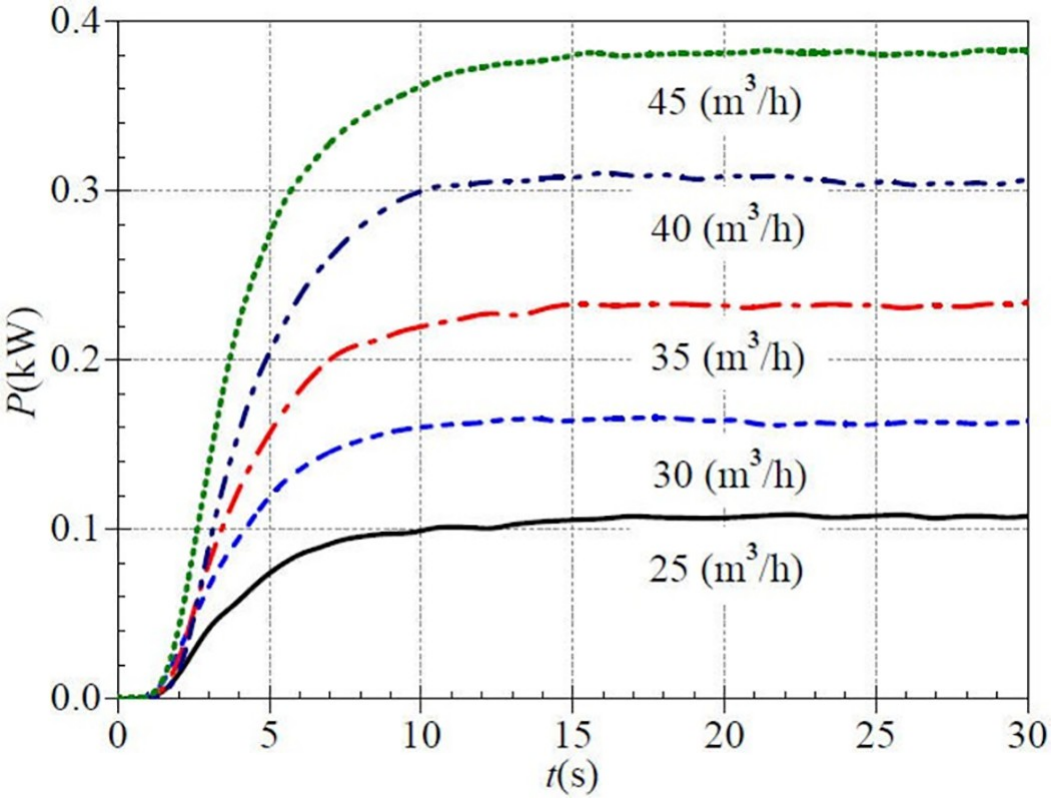
Transient shaft power rise (medium-speed start).

The shaft power curve also shows a general evolution of a slow rise, then a fast rise, and then a slow rise in Fig 13. The shaft power rises significantly after about 1.0 s, while it rises extremely slowly before 1.0 s. The rising rate differs for each valve opening case in the fast rise phase. At 5.0 s, the corresponding instantaneous shaft power is 0.074 kW, 0.119 kW, 0.157 kW, 0.205 kW, and 0.274 kW for the five valve openings, and the ratios of instantaneous shaft powers to their final steady values are 0.685, 0.726, 0.677, 0.670, and 0.719, respectively. It is seen that the rising rate is fastest in the lower middle opening and is slowest in the middle opening. After that, the shaft power curve slowly rises, and the final steady values are about 0.108 kW, 0.164 kW, 0.232 kW, 0.306 kW, and 0.381 kW, respectively. The higher the valve opening is, the greater the shaft power is. Comparing with the low-speed start, it is found that the higher the stable speed after the start, the higher the stable shaft power. However, the effect of valve opening on shaft power is more significant than that of rotational speed on shaft power.

### 3.4 High-speed start

**Fig 14:**
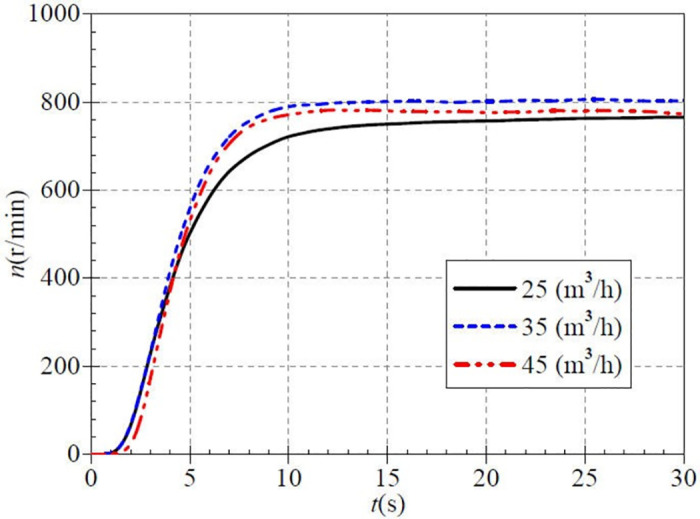
Rotational speed rise (high-speed start).

Fig 14 shows the rotational speed rise during high-speed start, corresponding to 25 m^3^/h, 35 m^3^/h, and 45 m^3^/h for the small, medium, and large valve opening cases. The three curves still have similar evolutionary characteristics to the low and medium-speed start. The rising rate of rational speed is highest in the medium opening, followed by the large opening, and is slowest in the small opening case. It can be found that the speed rise in the case of medium and large valve openings is significantly higher than that in the case of small valve openings. The main reason for this phenomenon is that because the speed rises more rapidly, the more significant flow rate causing torque is also greater. After about 20.0 s, the rotational speeds of the three valve open cases gradually reach the stable values of 755.836 r/min, 801.768 r/min, and 778.912 r/min, respectively. The measured stable rotational speeds still differ from the targeted values because of the unstable voltage during the experiment. The steady rotational speed value is maximum in the medium opening case and minimum in the small opening case.

**Fig 15:**
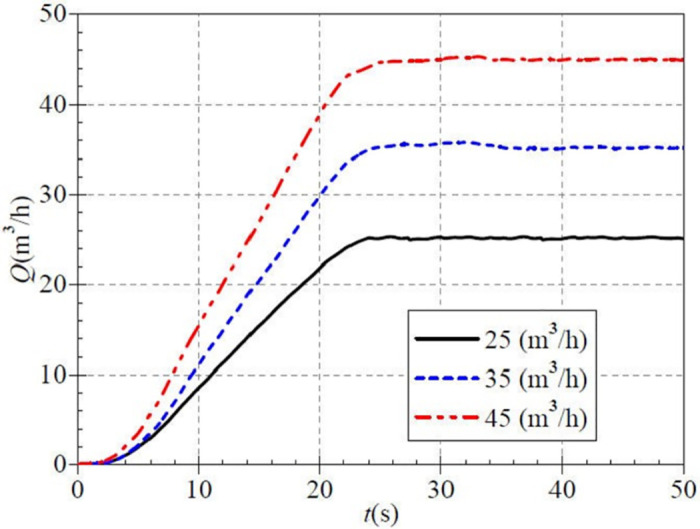
Transient flow rate rise (high-speed start).

Fig 15 shows that the measured steady flow rates for the three valve opening scenarios are 25.115 m^3^/h, 35.558 m^3^/h, and 44.836 m^3^/h, respectively, with minimal differences from the targeted values; this feature is consistent with the low and medium-speed startup. At 10.0 s, the flow rates are 8.589 m^3^/h, 11.123 m^3^/h, and 15.453 m^3^/h for the three openings, with ratios of 0.342, 0.313, and 0.345, respectively to the steady flow rate after startup. At 20.0 s, the flow rates are 21.857 m^3^/h, 29.831 m^3^/h, and 38.874 m^3^/h, whose ratios to the steady flow rate after startup are 0.871, 0.839, and 0.867, respectively. It can be seen that the individual flow rate increased by 49.7%, 52.6%, and 52.2%, respectively, in 10.0 s, which rose by nearly half during the 10.0 s time. At about 26.0 s, the instantaneous flow rate gradually increases to a steady value.

**Fig 16:**
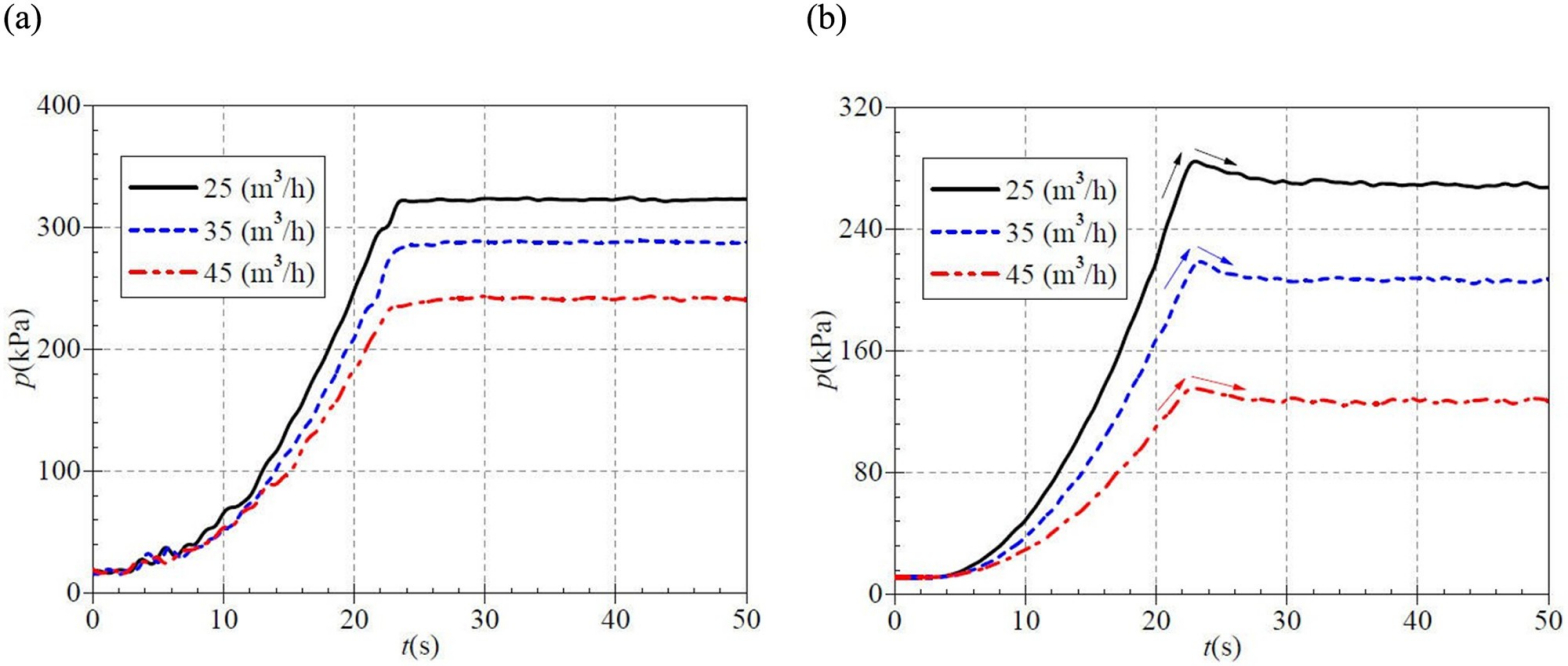
Transient static pressure rise (high-speed start) at (a) inlet (b) outlet.

Fig 16(a) shows that in the case of three valve openings, the corresponding stable static pressure at PAT inlet after startup are 322.4 kPa, 285.6 kPa, and 243.7 kPa, respectively, and the corresponding rise time is 23.6 s, 24.6 s, and 29.9 s. With the valve openings increasing, the atypical startup process shows a slight trend of delay in the time required for inlet static pressure to rise to stable value during the medium and high-speed start. Fig 16(b) shows that the outlet static pressure still has a shocking phenomenon during atypical startup. In the small, medium, and large valve opening cases, the measured maximum instantaneous static pressures are 284.821 kPa, 218.774 kPa, and 135.575 kPa, corresponding to the time of about 23.0 s, 23.4 s, and 22.8 s. It is found that the required time of shock static pressure is not very different. Thus, it can be seen that the required time of shock phenomenon at the outlet static pressure is not much related to the valve opening. The final static pressures after start are about 270.162 kPa, 205.882 kPa, and 128.728 kPa, and the shock static pressure is 14.659 kPa, 12.892 kPa, and 6.847 kPa, respectively, which shows that the static pressure shock phenomenon is the most intense in the case of small valve opening. With the increase of valve opening, the outlet static pressure’s impact phenomenon also shows a weakening trend. The comparison of the three starting processes shows that the strength of the static pressure shock is related to the start acceleration. The faster the start speed, the more intense the static pressure shock.

**Fig 17:**
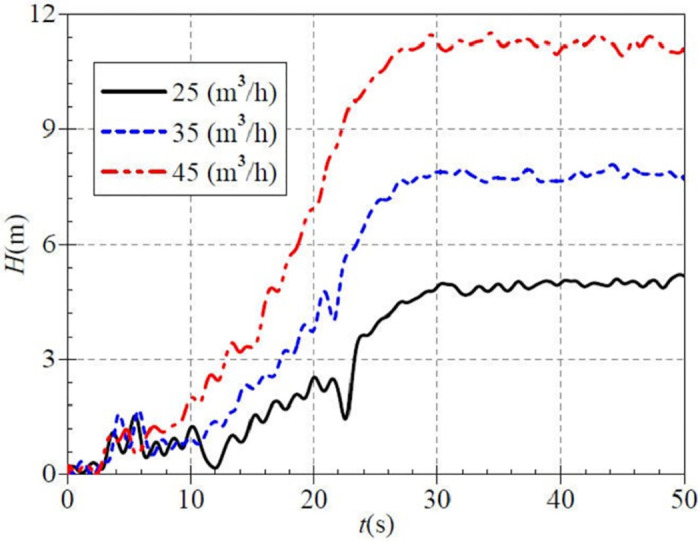
Transient head rise (high-speed start).

Fig 17 shows the average head after startup for the three valve openings of 4.95 m, 7.82 m, and 11.23 m. The comparative analysis o shows that as the steady rotational speed of PAT increases, the head also tends to grow, and its fluctuation is slowed down to some extent.

**Fig 18:**
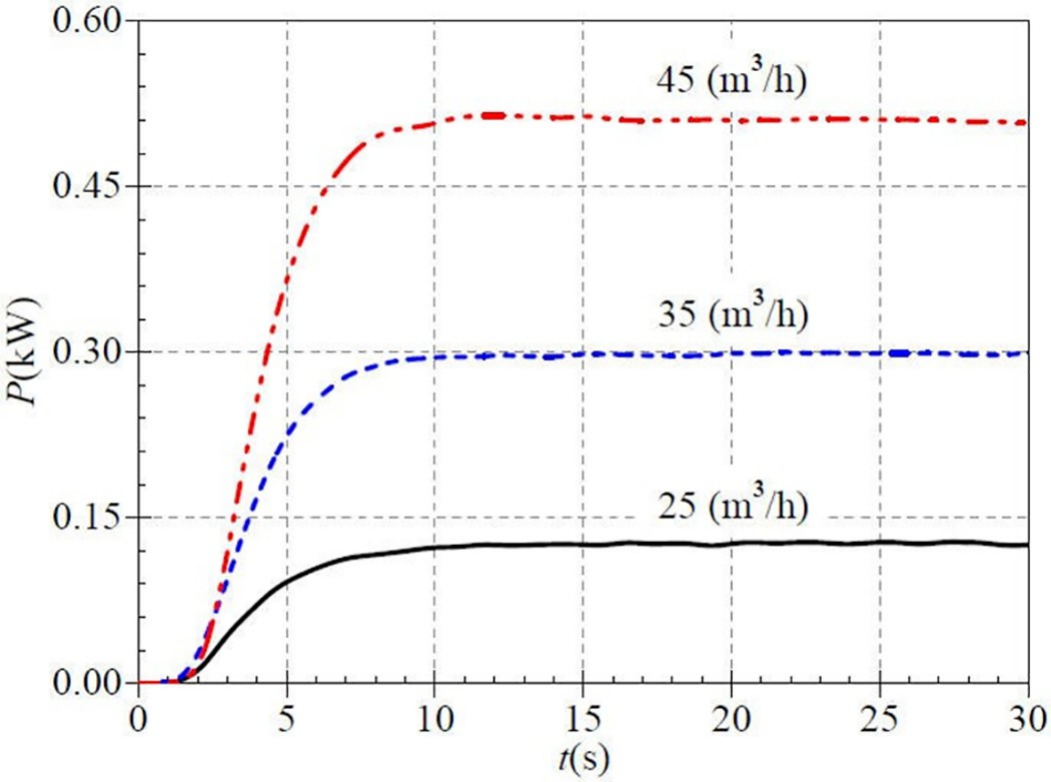
Transient shaft power rise (high-speed start).

Fig 18 shows that the shaft powers rise by 0.105 kW, 0.260 kW, and 0.478 kW from 2.0 s to 8.0 s after the start, which accounts for 82.7%, 87.2%, and 93.5% of the overall rise, respectively. The final stable values are 0.127 kW、0.298 kW, and 0.511 kW at about 12.0 s. It can be found that the effect of valve opening on shaft power is more significant compared to the effect of rotational speed value on shaft power.

### 3.5 Dimensionless analysis

The dimensionless volumetric flowrate, dimensionless head, and dimensionless shaft power are now used to further reveal the transient characteristics of PAT during atypical startup [28]. These three non-dimensional parameters are defined below.

$$\left\{\begin{array}{l} \phi \left(t\right)=Q\left(t\right)/\pi D_{2}b_{2}u_{2}\left(t\right)\\ \psi \left(t\right)=2gH\left(t\right)/u_{2}^{2}\left(t\right)\\ \Phi \left(t\right)=P\left(t\right)/\rho D_{2}^{2}u_{2}^{3}\left(t\right) \end{array}\right.$$

where *u*_2_(*t*) is the instantaneous peripheral speed at impeller outlet in pump operating mode, that is, the instantaneous peripheral speed at impeller inlet in turbine operating mode, which is expressed as *u*_2_(*t*) = π*D*_2_*n*(*t*)/60.

**Fig 19:**
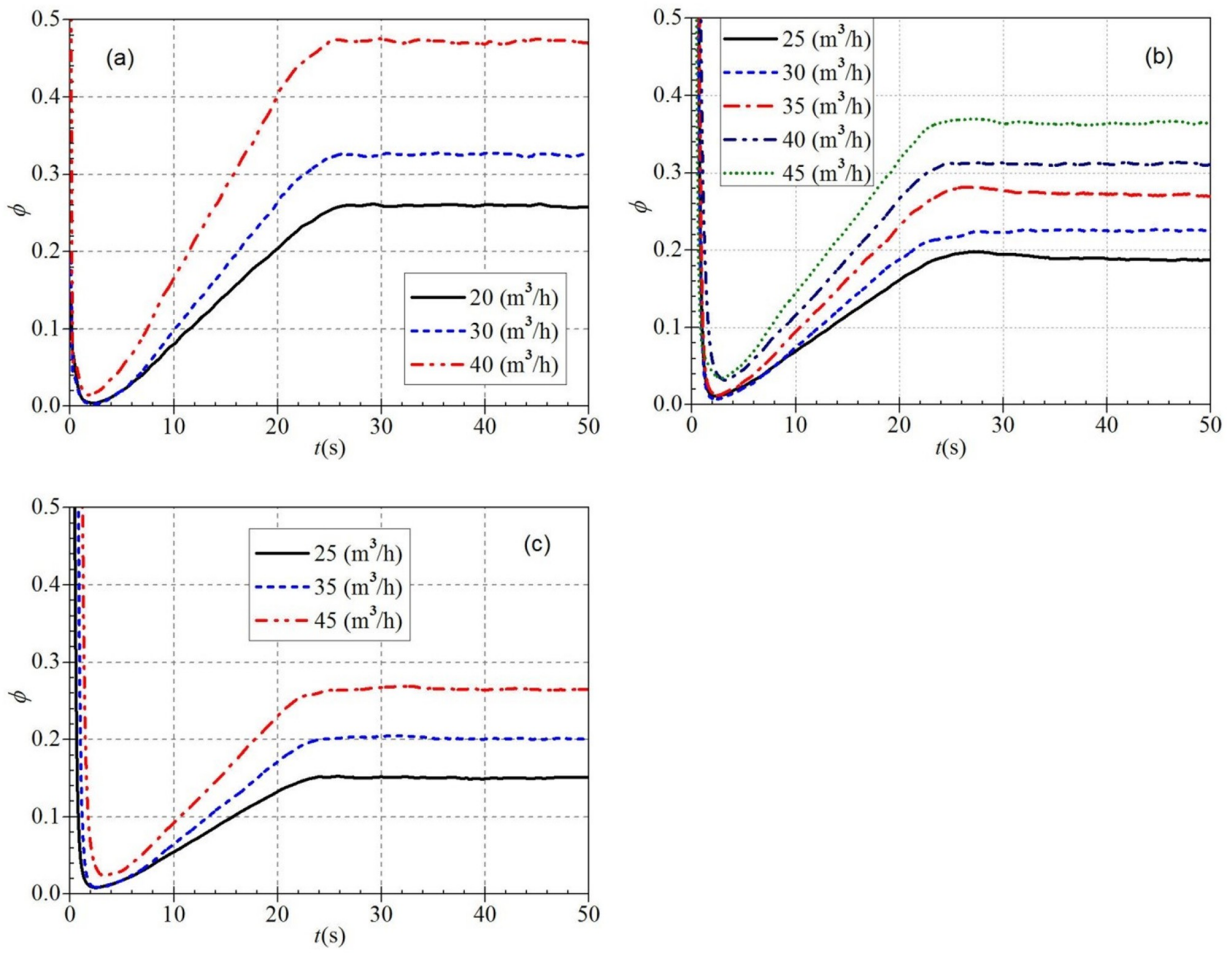
Dimensionless flow coefficients (a) low-speed start (b) medium-speed start (c) high-speed start.

It is seen from Fig 19 that the trends of the dimensionless flow coefficients during atypical startup are generally similar for any steady rotational speed and valve opening. At the beginning of startup, the dimensionless flow coefficients all have extreme values, then drop rapidly to a minimum value, then rise slowly to a final stable value. However, the time required to decrease from the extreme to the minimum value varies under different startup conditions. Fig 19(a) shows that the dimensionless flow coefficient drops to a minimal value at 2.3 s, 2.3 s, and 1.8 s, respectively. Subsequently, the three curves rise again to stable values at 27.1 s, 26.5 s, and 25.8 s. The corresponding stable values of the dimensionless flow coefficients are 0.260, 0.328, and 0.474, respectively. It can be seen that during the low-speed startup process, the dimensionless flow coefficient increases as the valve opening increases and the time to reach the stable value shifts forward. Fig 19(b) shows that the tremendous values at the beginning of the startup decrease to minimal values at 2.2 s, 2.3 s, 2.5 s, 3.3 s, and 2.9 s, respectively, and then they rise to stable values at about 34.5 s, 27.8 s, 31.4 s, 24.7 s, and 27.1 s.

Fig 19(c) shows that the minimum values are very close to the emergence times for the small and medium valve openings, reaching the minimum values at 2.5 s and 2.7 s, respectively. In contrast, the time to reach the minimum values for the large valve opening is first delayed compared to the first two valve openings, reaching the minimum values at 3.3 s. Then the three curves rise slowly and reach stable values at 24.0 s, 27.0 s, and 31.7 s, respectively. The required time to reach the stable value lags as the valve opening increases during the high-speed startup.

Combining the above three startup statuses, it can be found that both valve opening and steady rotational speed are essential factors in determining the variation characteristics of the dimensionless flow coefficient. The steady rotational speed variation mainly influences the dimensionless flow coefficient. The corresponding dimensionless flow coefficients are about 0.272 and 0.201 when the startup ends in regular and steady operation, respectively. It can be seen that the dimensionless flow coefficient shows a gradually decreasing trend with the increase of the steady rotational speed after the startup, and the dimensionless flow coefficient shows a gradually increasing trend with the rise of the steady flow rate.

**Fig 20:**
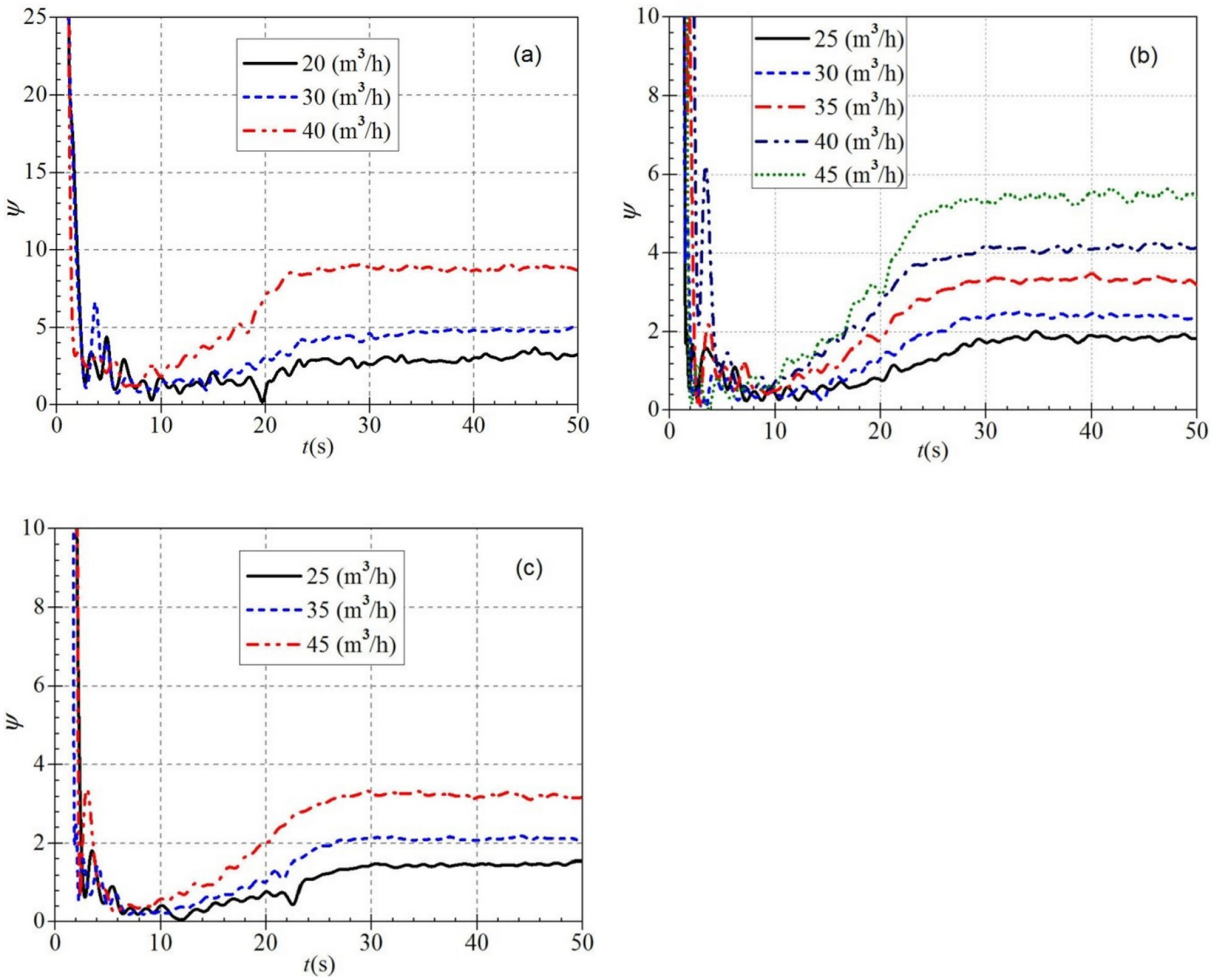
Dimensionless head coefficient (a) low-speed start (b) medium-speed start (c) high-speed start.

By comparing Figs 19 and 20, it can be found that the dimensionless head coefficients and flow coefficients generally have approximately the same evolutionary trend during the atypical start-up process. However, there are still some differences between them; the dimensionless head coefficient changes more drastically than the dimensionless flow rate coefficient in the same situation. It can be seen that the dimensionless head coefficient also shows a gradually decreasing trend with the increase of the stable rotational speed after starting. At the same time, it also shows a gradually increasing trend with the rise of the stable flow rate after starting.

**Fig 21:**
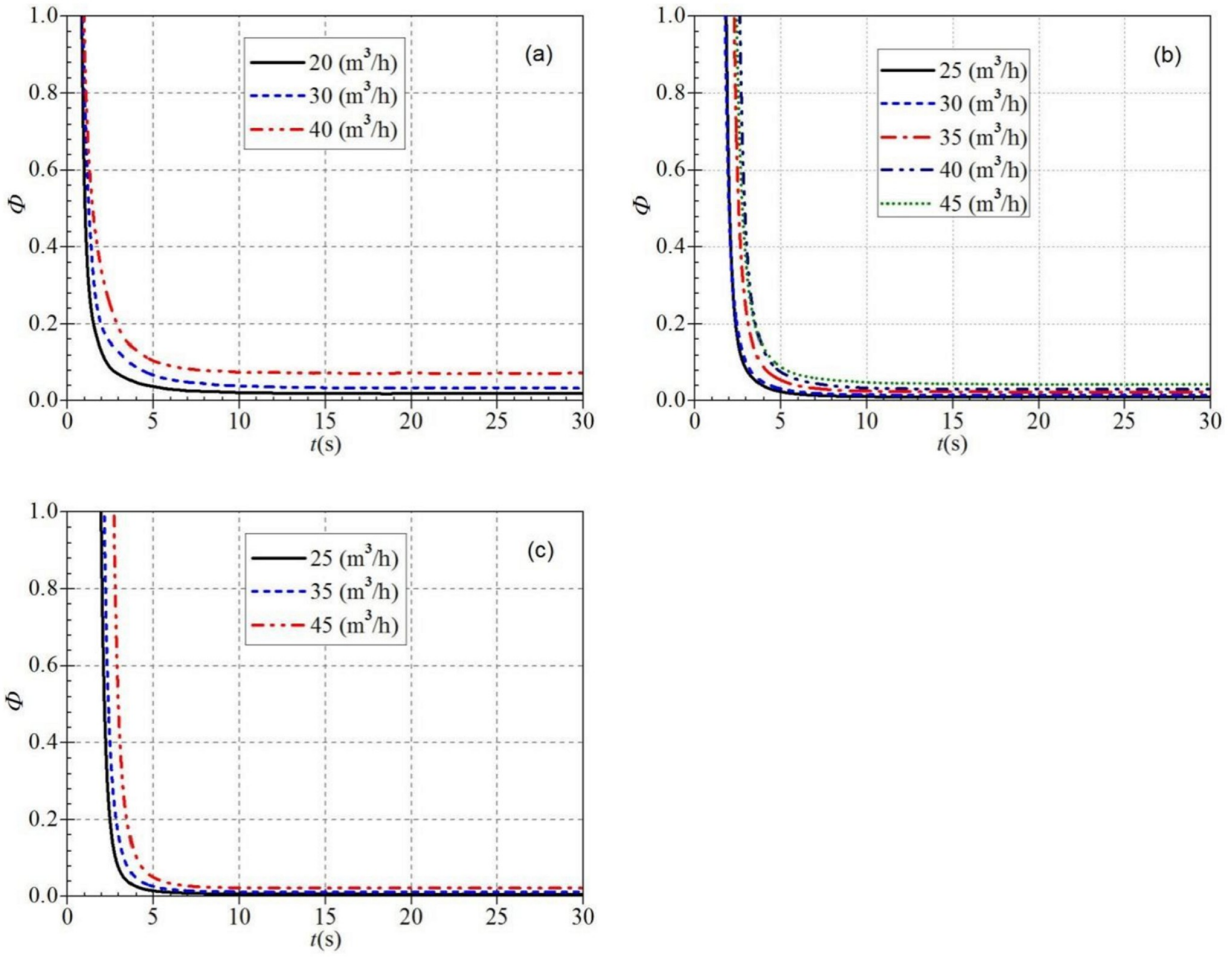
Dimensionless power coefficient (a) low-speed start (b) medium-speed start (c) high-speed start.

It is seen from Fig 21 that during the atypical startup of PAT, the dimensionless power coefficient has a very different evolution trend from the dimensionless flow rate and head coefficient, namely that the dimensionless power coefficient has a great value at the beginning of the atypical startup, and then decreases rapidly to the final stable value. With the increase of stable flow rate after startup, the dimensionless power coefficient also shows a gradual increase, but its increase is negligible compared with the other two dimensionless coefficients.

### 3.6 Nominal acceleration time

The valve opening affects the final stabilization value and the acceleration time of the entire startup process during the atypical startup. It is seen that the time required for each performance parameter to reach stability is different during the startup process. To better analyze the effect of valve opening on the startup process, a nominal acceleration time is introduced [29,30], defined as the time required for each performance parameter to reach its stable value of 63.2%. By comparing the nominal acceleration time for each parameter at different valve openings, the influence degree of the valve opening on each parameter can be more intuitively reflected.

**Fig 22:**
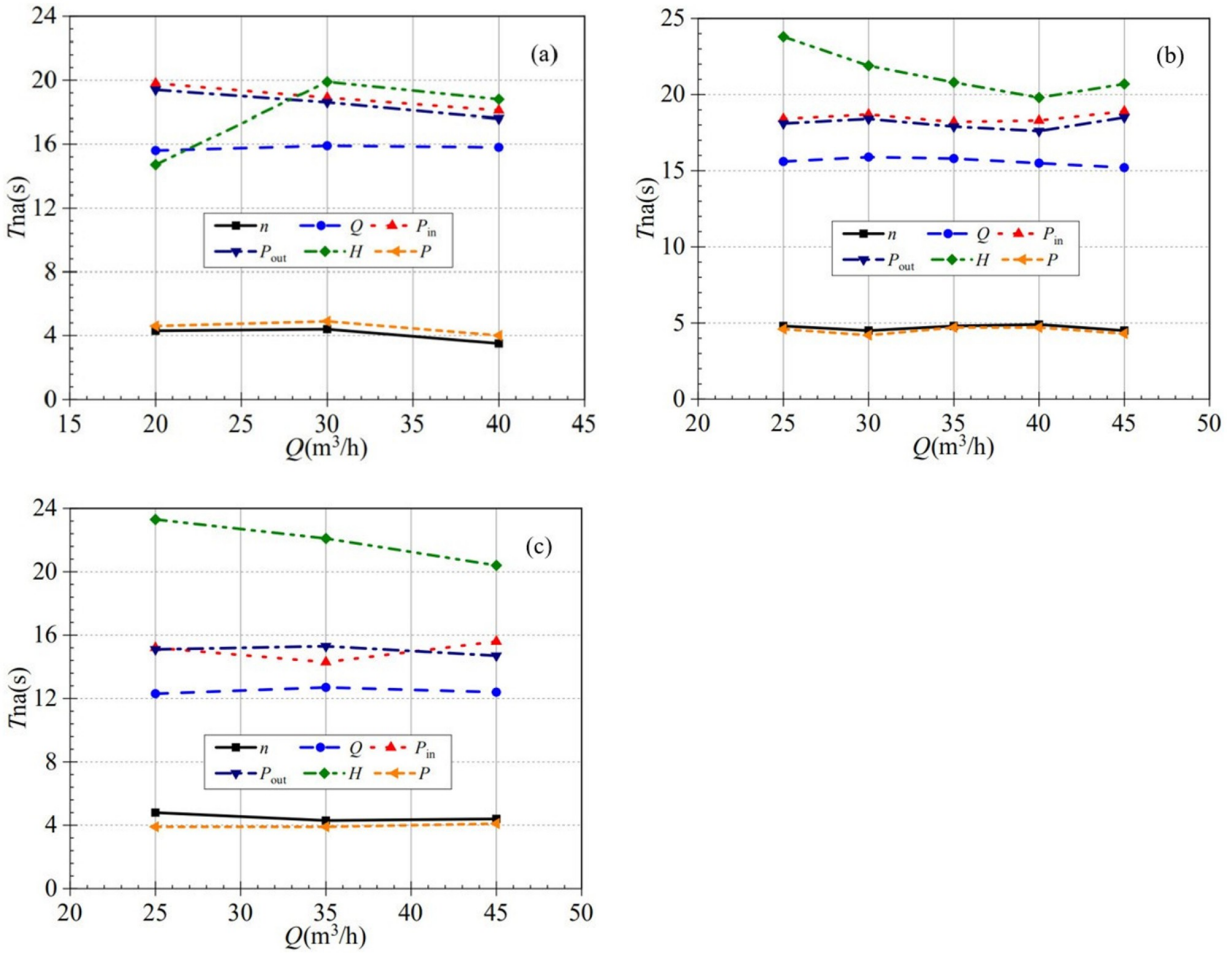
Nominal acceleration time (a) low-speed start (b) medium-speed start (c) high-speed start.

Fig 22 shows that the nominal acceleration time of the shaft power and the rotational speed is much smaller than the other performance parameters, i.e., the rising rate of the shaft power and the rotational speed is much faster than the rest of the performance parameters during the startup process. In the process of low-speed start, the curves of shaft power and rotational speed both show a rising and decreasing trend with the increase of valve opening. The nominal acceleration times of shaft power at the three valve openings were 4.6 s, 4.9 s, and 4.0 s, with growth rates of 6.52% and -18.37%, respectively. Meanwhile, the nominal acceleration times of rotational speed are 4.3 s, 4.4 s, and 3.5 s, with growth rates of 2.32% and -20.45%, respectively. In the process of medium-speed start, the shaft power and rotational speed curves still show similar trends, both decreasing, then increasing, and then decreasing. As demonstrated in Fig 22, the two curves show a high degree of overlap. The nominal acceleration times for shaft power at the five valve openings are 4.6 s, 4.2 s, 4.7 s, 4.7 s, and 4.3 s, with growth rates of -8.69%, 11.91%, 0%, and -8.51%, respectively, while the nominal acceleration times for rotational speed are 4.8 s, 4.5 s, 4.8 s, 4.9 s, and 4.5 s, with growth rates of -6.25%, 6.67%, 2.08%, and -0.082%, respectively. In the process of high-speed start, the shaft power shows a rising trend, while the rotational speed shows a falling and then rising trend, and the rotational speed change is much more drastic than the change of shaft power. The nominal acceleration times for shaft power are 3.9 s, 3.9 s, and 4.1s, with 0% and 5.13% growth rates for the three valve openings, respectively. The nominal acceleration times for speed are 4.8 s, 4.3 s, and 4.4 s, with -10.42% and 2.33% growth rates, respectively. It can be seen that both shaft power and rotational speed show highly similar characteristics in terms of growth trend and nominal acceleration time, and the size of the valve opening has a specific influence on the acceleration time. In addition, the rising rate of shaft power is relatively fastest at a high-speed start, while the rotational speed is relatively fastest at low-speed startup.

Similarly, the nominal acceleration time of the pressures at the inlet and outlet has highly similar characteristics. For the low-speed start, the nominal acceleration time shows a decreasing trend with increasing valve opening. At the three valve openings, the nominal acceleration times for inlet pressure are 19.8 s, 18.9 s, and 18.1 s with growth rates of -4.54% and -4.23%, respectively, while the nominal acceleration times for outlet pressure are 19.4 s, 18.6 s, and 17.6 s with growth rates of -4.12% and -5.38%, respectively. For the medium-speed start, the pressure curves at the inlet and outlet show a rising trend, then falling, then rising. The nominal acceleration times of inlet pressure at five valve openings are 18.4 s, 18.7 s, 18.2 s, 18.3 s, and 18.9 s, with growth rates of 1.63%, -2.67%, 0.55%, and 3.28%, respectively, while the nominal acceleration times of outlet pressure are 18.1 s, 18.4 s, 17.9 s, 17.6 s, and 18.5 s, with growth rates of 1.66%, -2.72%, -1.68%, and 5.11%. For the high-speed start, the inlet pressure curve shows a decreasing trend followed by an increasing trend, while the outlet pressure curve shows the opposite trend. The nominal acceleration times of inlet pressure at the three valve openings are 15.2 s, 14.3 s, and 15.6 s, with growth rates of -5.92% and 9.01%, respectively, while those at the outlet are 15.2 s, 14.3 s, and 15.6 s, with growth rates of 1.32% and -3.92%, respectively. It can be found that the nominal acceleration time changes with the change of valve opening under the same starting condition, and the trend of change is not the same under different acceleration conditions. Unlike the shaft power and rotational speed curves, the difference in nominal acceleration time between medium-speed and high-speed is more prominent. As can be seen, the rising rate at which the inlet and outlet pressures reach stable values is much faster for the high-speed start than for medium-speed start.

The nominal acceleration time curve corresponding to the flow rate is relatively stable at different valve openings. The nominal acceleration time does not vary much between different valve openings at each startup condition. Consistent with the pressures at the inlet and outlet, the nominal acceleration time of the flow rate decreases significantly during the high-speed start. For example, in the targeted stable flow rate of 35 m^3^/h, the nominal acceleration time of the flow rate in the medium-speed start condition is 15.8 s, while it in the high-speed start condition drops to 12.7 s, with a difference of 3.1 s. It can be seen that the flow rate in the medium and high-speed start conditions, the acceleration difference is more significant; that is, the high-speed start than the medium-speed start acceleration to the stable flow rate is much faster. Unlike other performance parameter curves, the head curve shows different trends under different starting conditions. The head curve increases and decreases as the valve opening increases for the low-speed start. The nominal acceleration time of heads are 14.7 s, 19.9 s, and 18.8 s, respectively. The nominal acceleration time from small valve to medium valve opening is considerable, with a growth rate of 35.37%. During the medium-speed startup, the nominal acceleration curves of head show a trend of decreasing and then increasing, with nominal acceleration times of 23.8 s, 21.9 s, 20.8 s, 19.8 s, and 20.7 s at the five valve openings, with growth rates of -7.98%, -5.02%, -4.81%, and 4.54%, respectively. During the high-speed start, the nominal acceleration curves of head show a trend of decreasing all the time with nominal acceleration times of 23.3 s, 22.1 s, and 20.4 s, and its growth rates are -5.15% and -7.69%, respectively.

In summary, the rising rate of the shaft power and rotational speed is similar. Moreover, the rising rate of rising to stable conditions is also significantly faster than the other parameters. The nominal acceleration time of flow rate is less evident with the change of valve opening. In contrast, the nominal acceleration time of head changes drastically with the change of valve opening, and the maximum difference between adjacent valve openings reaches 5.2 s.

In order to better understand the rising rate of performance parameters at different startup processes, a dimensionless time (*λ*) is introduced, which is defined as the ratio of the nominal acceleration time (*T*_na_) to the total time (*T*_0_) required for the parameter itself to reach stability. The ratio fully reflects the rising rate of each parameter in the first half of the startup process, and a smaller ratio means that each performance parameter rises faster in the first half of the startup process and slower in the second half, and vice versa.


$$\lambda =T_{\text{na}}/T_{0}$$

**Fig 23:**
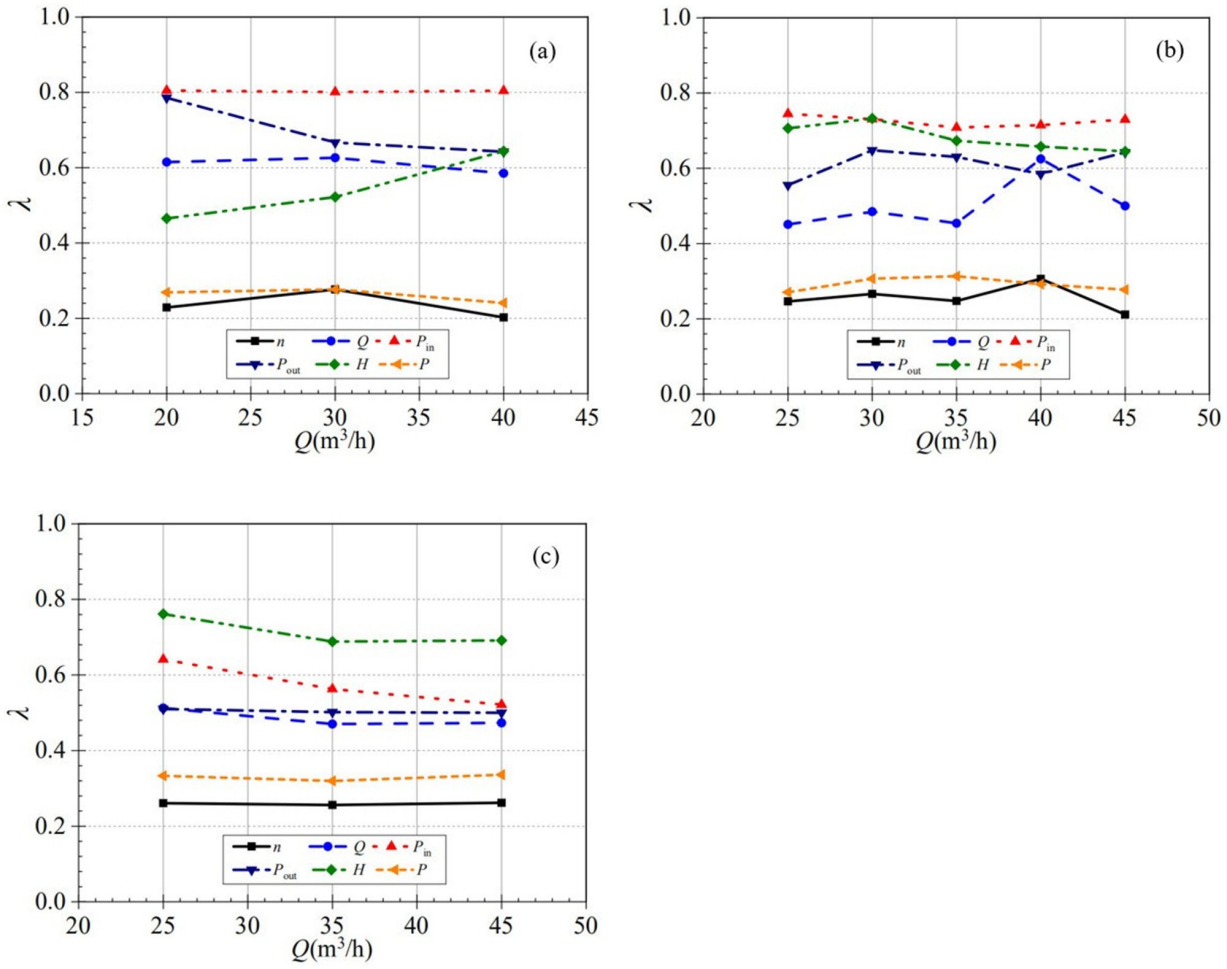
Dimensionless time (a) low-speed start (b) medium-speed start (c) high-speed start.

Fig 23 shows that the dimensionless time can fully reflect the rising speed in the first half of the starting process, and the smaller its value, the faster the rising speed in the first half. It can be found that the dimensionless time corresponding to shaft power and rotational speed fluctuates around 0.3, i.e., the rising rate of shaft power and rotational speed in the first half of the starting process is much larger than that in the second half. Unlike the nominal acceleration time, the inlet and outlet static pressure curves are not so similar in dimensionless time. For example, at the low-speed start, the dimensionless times of inlet static pressure are 0.805, 0.801, and 0.804, which are extremely small, while in contrast, the dimensionless times for the outlet static pressure were 0.785, 0.667, and 0.642, respectively, with a difference of 0.118 between the small and medium valves. This difference may be due to the pressure shock phenomenon of the outlet static pressure during the startup. Since the oscillation change of the head is extremely obvious during the startup process, the dimensionless time changes drastically at different valve openings. For the low-speed start, the dimensionless time rises and then drops with the rising of the valve opening. The dimensionless time is 0.525, 0.522, and 0.644 at three valve openings. The large valve opening first decreases by 0.003 and then increases by 0.122, i.e., the head starts fastest in the first half of the process at the medium valve opening. During the medium-speed startup, the dimensionless time of the flow rate at the upper middle valve opening (40 m^3^/h) is extraordinary; its value increases abruptly from 0.454 to 0.625 from the middle valve opening and decreases gradually to 0.5. The reason for this situation is that, unlike the flow rate values at other valve openings, the shocking phenomenon of flow rate does not occur at the upper middle valve opening, which leads to its time to reach the stable working condition earlier, which in turn leads to an increase in the dimensionless time.

In summary, the shaft power and the rotational speed rise very fast in the first half of the startup process, while the inlet pressure rises the slowest in the first half, and the difference between them is several times. In the low-speed start process, the valve opening has a pronounced effect on the dimensionless head time; the more significant the valve opening, the slower the rise in the first half of the startup process. In the medium-speed start process, the dimensionless time of the head decreases with the increase of the valve opening. It can be seen that the dimensionless time of head is not directly related to the valve opening. Unlike the nominal acceleration time, the valve opening affects the dimensionless time of flow rate, and it is most evident in the low-speed start process.

### 3.7 Discussion

In this experiment, three to five flowrate conditions are selected for comparative analysis at each steady rotational speed by changing the outlet valve opening of PAT at three steady rotational speeds (400 r/min, 600 r/min, 800 r/min) to investigate the effect of flowrate and rotational speed on the transient hydraulic performance of PAT during the atypical start-up process. It can be seen that the differences between steady rotational speeds and steady flow rates are small, especially the latter. Therefore, it is the next work to systematically explore the effect of more significant rotational speed differences and flowrate differences on transient hydraulic performance in future work.

## 4 Conclusion

The rising rate of the shaft power and the rotational speed of PAT is the highest, followed by flow rate and inlet and outlet pressure, and finally head. The shaft power and rotational speed rise extremely fast in the first half of the startup process, while the rise of inlet pressure is the slowest in the first half of the startup process. The evolution trends of dimensionless flow coefficient and dimensionless head coefficient are similar, while the evolution trends of both of them are very different from dimensionless power coefficient. As the valve opening increases, the dimensionless flowrate, head, and power coefficients all show a gradual increase in evolution. The outlet static pressure of PAT generally has a shocking phenomenon. The research in this paper has practical significance for optimizing the operation of power plants and industrial facilities. Accurately adjusting the valve opening can effectively control the pump and turbine’s startup speed, improving the operation efficiency and extending equipment life. Additionally, a better understanding of these characteristics can aid in selecting and designing equipment and pipe systems, which can reduce material costs and ensure that the system operates safely and reliably.
